# Progress and prospects of applying carbon‐based materials (and nanomaterials) to accelerate anaerobic bioprocesses for the removal of micropollutants

**DOI:** 10.1111/1751-7915.13822

**Published:** 2021-09-29

**Authors:** Ana Rita Silva, Maria Madalena Alves, Luciana Pereira

**Affiliations:** ^1^ CEB –Centre of Biological Engineering University of Minho Campus de Gualtar Braga 4710‐057 Portugal

## Abstract

Carbon‐based materials (CBM), including activated carbon (AC), activated fibres (ACF), biochar (BC), nanotubes (CNT), carbon xenogels (CX) and graphene nanosheets (GNS), possess unique properties such as high surface area, sorption and catalytic characteristics, making them very versatile for many applications in environmental remediation. They are powerful redox mediators (RM) in anaerobic processes, accelerating the rates and extending the level of the reduction of pollutants and, consequently, affecting positively the global efficiency of their partial or total removal. The extraordinary conductive properties of CBM, and the possibility of tailoring their surface to address specific pollutants, make them promising as catalysts in the treatment of effluents containing diverse pollutants. CBM can be combined with magnetic nanoparticles (MNM) assembling catalytic and magnetic properties in a single composite (C@MNM), allowing their recovery and reuse after the treatment process. Furthermore, these composites have demonstrated extraordinary catalytic properties. Evaluation of the toxicological and environmental impact of direct and indirect exposure to nanomaterials is an important issue that must be considered when nanomaterials are applied. Though the chemical composition, size and physical characteristics may contribute to toxicological effects, the potential toxic impact of using CBM is not completely clear and is not always assessed. This review gives an overview of the current research on the application of CBM and C@MNM in bioremediation and on the possible environmental impact and toxicity.

## Graphical abstract

Carbon nanomaterials are powerful redox mediators in anaerobic processes accelerating the treatment of effluents containing different pollutants. The proper characteristics of the various nanomaterials have advantages and disadvantages in relation to each other. Evaluation of the toxicological and environmental impact of direct and indirect exposure to nanomaterials is an important issue.

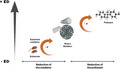

## Introduction

Nanomaterials (NM) are interesting for environmental remediation purposes due to their high surface area, enhancing the interactions with the contaminant, their small size, enabling their penetration or diffusion in contaminated areas and bioreactors, and their high reactivity to redox‐amenable contaminants (Pereira, *et al*., 2015; Santhosh *et al*., 2016). A wide range of NM, including nanoscale zeolites, bimetallic nanoparticles, metal oxides and CBM (e.g. AC, ACF, CNT and GO), has been proposed as nanosorbents and nanocatalysts on chemical and biological processes for the bioremediation of different pollutants (van der Zee *et al*., 2003; Gonçalves *et al*., 2010; Mezohegyi *et al*., 2012; Orge *et al*., 2012; Pereira *et al*., 2014; Patil *et al*., 2016; Pereira et al., 2016b; Santhosh *et al*., 2016; Ahsan *et al*., 2020).

Some of these contaminants are considered micropollutants (MP), as they appear in water effluents at very low concentrations, ranging from μg l^‐1^ to ng l^‐1^ (Eggen *et al*., 2014; Luo *et al*., 2014; Gonzalez‐Gil *et al*., 2016; Subedi and Loganathan, 2016), but tend to accumulate and persist in water bodies leading to adverse environmental effects, in particular short‐term and long‐term toxicity of microflora and fauna (Pereira, 2014). MP can even enter the food chain and ultimately reach humans. Some of them, e.g. pharmaceutical compounds, can cause endocrine‐disrupting effects and increase bacteria resistance (Eggen *et al*., 2014; Stamm *et al*., 2016; Pazda *et al*., 2019).

Wastewater treatment plants (WWTP) represent a potential primary barrier against the spreading of MP (Grandclément *et al*., 2017; Krzeminski *et al*., 2019). However, conventional WWTP are not designed for the removal of these specific recalcitrant compounds, and most of them pass through the processes or adsorb on the sludge, being continuously introduced in the environment (Luo *et al*., 2014; Bui *et al*., 2016; Dong *et al*., 2016; Rizzo *et al*., 2019). In addition to the recalcitrant nature of MP, their presence in very low concentrations represents a major limitation for the removal in the WWTP (Gonzalez‐Gil *et al*., 2016; Subedi and Loganathan, 2016). MP concentrations are orders of magnitude lower than other carbon sources typically found in domestic wastewater and are not a primary carbon source for the microorganisms (Fischer and Majewsky, 2014; Harb *et al*., 2019). In this sense, the environmental problem related to water pollution by MP has received special attention from the World Health and Environmental Organizations, which have been developing more effective policies for wastewaters control and for a sustainable exploitation of water resources (World Health Organization; United Nations Environment Programme, 1997). Yet, the discharge limits of these compounds and their transformation products still remain not regulated, mainly because they appear in concentrations below the usual environmental quality standards (Barbosa *et al*., 2016; Kümmerer *et al*., 2019). Thus, the prevention and elimination of these compounds is an urgent need and a challenge for the scientific community (Kümmerer *et al*., 2019; Rizzo *et al*., 2019).

Biological removal of several MP under anaerobic conditions, occurring through reductive reactions, where the pollutant is the final electron acceptor, has been reported (Healy and Young, 1979; Pereira et al., 2016b; Ghattas *et al*., 2017; Völker *et al*., 2017). However, reductive reactions proceed slowly due to the recalcitrant nature of these compounds and to electron transfer limitations. This is an hindrance for its application in high‐rate anaerobic bioreactors, because of the need of long hydraulic retention times (HRT) to attain a reasonable degree of pollutant reduction (van der Zee *et al*., 2003; Stasinakis, 2012; Pereira et al., 2016b; Dubey *et al*., 2021). Nevertheless, anaerobic biodegradation can be accelerated by applying redox mediators (RM), compounds that act as electron shuttles in multiple redox reactions between the microorganisms and the MP, so increasing the global reaction rates by lowering the corresponding activation energy (van der Zee et al., 2001a,2001b; van der Zee and Cervantes, 2009). The efficacy of RM is directly related with their properties, being the standard redox potential (E0′) and activation energy key aspects to consider. Ideally, RM should decrease the reaction’s activation energy, so their E0′ has to be in between the primary electron donor and the final electron acceptor, for instance the pollutant being reduced (Cervantes and dos Santos, 2011).

Non‐soluble materials, like CBM, have demonstrated singular properties as RM in chemical and biological processes (van der Zee, Bouwman, *et al*., 2001; Mezohegyi *et al*., 2012; Baêta *et al*., 2013). Besides the significant improvements on reaction rates, which will be discussed next, these materials can be immobilized in the reactors and reused several times, so being an attractive alternative to soluble RM that require continuous addition and are released with the treated effluent (Pereira et al., 2016b).

## Carbon‐based materials (and nanomaterials) as redox mediators

Several non‐soluble CBM, including AC (granular, powder, fibres) (van der Zee *et al*., 2003; Pereira *et al*., 2010; Amezquita‐Garcia *et al*., 2016; Dai *et al*., 2016), BC (Kappler *et al*., 2014; Tong *et al*., 2014), CNT (Pereira *et al*., 2014; Pereira *et al*., 2016), CX (Pereira *et al*., 2014; Pereira et al., 2016a) and GNS (Wang *et al*., 2014; Li *et al*., 2016), have been used as RM in the biological degradation of different pollutants. Comparing to soluble RM, CBM can be retained within the sludge bed and so can be immobilized in bioreactors, which is a remarkable advantage in terms of efficiency and also costs (Mezohegyi *et al*., 2007, 2008, 2012; González‐Gutiérrez *et al*., 2009; Butkovskyi *et al*., 2018).

Table 1 summarizes some studies reporting the influence of various CBM, performing as RM, on the biological removal of contaminants from water, in batch and continuous reactors, by the action of different microorganisms and substrates. The different characteristics of each CBM, including the textural properties like the total specific surface area (S_BET_), the non‐microporous surface area (Smeso) and the porous size (micro‐, meso‐ or macroporous), influence their performance as RM (Table S1). CBM have a highly porous structure which confers them a high surface area. They have also a chemical structure with a wide range of active sites, allowing its interaction with several molecules of different nature (Pereira *et al*., 2014; Wang *et al*., 2014; Pereira et al., 2016a).

**Table 1:** Effect of carbon nanomaterials and composite carbon nanomaterials, as redox mediators (RM), on the biological reduction of organic pollutants.

RM	Pollutant	Microorganisms			Experimental setup	Electron donor	RM concentration (g l^‐1^)	Pollutant removal %	Degradation rate (day^‐1^)	References
AC	HRR2–0.073 mM	AGS – 35 g l^‐1^ VSS			UASB reactors (250 ml – WV)	VFA (acetic, propionic and butyric acid, 1:1:1) – 1.5 g l^‐1^ COD	c.a.	40	0.13	van der Zee *et al*. (2003)
							2.5	97	0.31	
							0.1	78	0.25	
	AO 7–0.28 mM	AS ‐ 1 g ml^‐1^			UPBR (9 ml – WV)	Sodium acetate – 0.2 g l^‐1^	c.a.	n.d.	n.d.	Mezohegyi *et al*. (2007)
							110	99	74.0	
					USPBR (10 ml – WV)	Sodium acetate – 0.2 g l^‐1^	c.a.	n.d.	n.d.	Mezohegyi *et al*. (2008)
							100	96	278	
	Reactive red (RR) 272–0.51 mM	AS – 11.586 mg g^‐1^ SS			UAFBR (3 l – WV)	Dextrose; yeast extract	c.a.	n.d.	n.d.	González‐Gutiérrez *et al*. (2009)
							436	97	3.37	
	MY10 – 0.3 mM	AGS – 1 g l^‐1^ VSS			Batch anaerobic reactors	VFA (acetic, propionic and butyric acid, 1:10:10) – 2 g l^‐1^	c.a.	87 ± 1	10.2 ± 1.7	Pereira *et al*. (2010)
							0.6	78 ± 1	11.3 ± 1.2	
							0.4	83 ± 2	9.8 ± 2.2	
							0.1	86 ± 1	10.2 ± 1.4	
	Methyl red (MR) – 0.2 mM.	AGS – 0.5 g l^‐1^ VSS			Batch anaerobic reactors (60 ml – NV)	Glucose – 3 g l^‐1^	c.a.	32	0.237 ± 0.006	Emilia Rios‐Del Toro et al. (2013)
							0.2d	80	0.875 ± 0.006	
	o‐NoA – 1 mM	AGS – 2.5 ± 0.5 g l^‐1^ VSS			Batch anaerobic reactors (25 ml – WV)	VFA (acetic, propionic and butyric acid, 1:10:10) – 2 g l^‐1^ COD	c.a.	32 ± 1	0.07 ± 0.01	Pereira *et al*. (2016)
							0.1	97 ± 2	3.6 ± 0.5	
	m‐NoA – 1 mM	AGS – 2.5 ± 0.5 g l^‐1^ VSS			Batch anaerobic reactors (25 ml – WV)	VFA (acetic, propionic and butyric acid, 1:10:10) – 2 g l^‐1^ COD	c.a.	56 ± 4	0.26 ± 0.11	
							0.1	98 ± 1	27.36 ± 1	
	p‐NoA – 1 mM	AGS – 2.5 ± 0.5 g l^‐1^ VSS			Batch anaerobic reactors (25 ml – WV)	VFA (acetic, propionic and butyric acid, 1:10:10) – 2 g l^‐1^ COD	c.a.	52 ± 2	0.14 ± 0.02	
							0.1	97 ± 1	25.2 ± 0.24	
	AO10 – 0.50 mM	AGS – 10 g l^‐1^ VS			UASB reactor (400 ml – WV)	VFA (acetic, propionic and butyric acid, 1:10:10) – 2 g l^‐1^ COD	c.a.	16 ± 4	0.38	Pereira *et al*. (2016)
							1.2	90 ± 2	2.2	
	Congo Red (CR) – 0.22 mM	AGS – 0.1 g l^‐1^ VSS			Batch anaerobic reactors (50 ml – WV)	Glucose – 1 g l^‐1^	c.a.	20 ± 1.8	0.24 ± 0.01	Alvarez *et al*. (2017)
							1.13	25 ± 7.8	0.33 ± 0.01	
	CR – 0.14 mM	AGS – 5 g l^‐1^ VSS			UASB reactor (190 ml – WV)	Glucose – 1 g l^‐1^ COD	9	57 ± 4.4	0.40	
						Glucose; *p*‐cresol – 0.6 g l^‐1^ COD	9	71 ± 8.4	0.24	
	Ibuprofen – 0.00076 mM						c.a.	30	1.1 x10^‐5^	Butkovskyi *et al*. (2018)
		FS – 5:1 COD‐based mixture of black water and sludge from grey water treatment system			UASB reactor (4.7 l – WV)	n.d.	5.7	60	2.3 x10^‐5^	
	Diclofenac – 0.00046 mM						c.a.	60	1.4 x10^‐5^	
							5.7	67	1.5 x10^‐5^	
	Metoprolol – 0.00038 mM						c.a.	10	1.9 x10^‐6^	
							5.7	70	1.3 x10^‐5^	
	Galaxolide – 0.0013 mM						c.a.	20	1.3 x10^‐5^	
							5.7	75	4.9 x10^‐5^	
	Triclosan – 0.00040 mM						c.a.	40	8.0 x10^‐6^	
							5.7	80	1.6 x10^‐5^	
	Municipal sewage – 0.5 g l^‐1^ COD	AS‐ 3L			UASB reactor (4.7 l – WV)	n.d.	c.a.	56	0.28	Zhang et al. (2020a,b)
							25	82	1.64	
AC _H2_	MY10 – 0.3 mM	AGS – 1 g l^‐1^ VSS			Batch anaerobic reactors	VFA (acetic, propionic and butyric acid, 1:10:10) – 2 g l^‐1^	c.a.	87 ± 1	10.2 ± 1.7	Pereira *et al*. (2010)
							0.6	89 ± 1	19.6 ± 1.5	
							0.4	88 ± 0	23.6 ± 3.8	
							0.1	87 ± 1	19.4 ± 0.2	
	MY10 – 1 mM	AGS – 2.5 ± 0.5 g l^‐1^ VSS			Batch anaerobic reactors (25 ml – WV)	VFA (acetic, propionic and butyric acid, 1:10:10) – 2 g l^‐1^ COD	c.a.	83 ± 1	9.50 ± 0.49	Pereira *et al*. (2014)
							0.1	85 ± 1	11.02 ± 0.7	
	RR120 – 1 mM	AGS – 2.5 ± 0.5 g l^‐1^ VSS			Batch anaerobic reactors (25 ml – WV)	VFA (acetic, propionic and butyric acid, 1:10:10) – 2 g l^‐1^ COD	c.a.	67 ± 3	3.09 ± 0.30	
							0.1	68 ± 3	3.14 ± 0.04	
	AO10 – 1 mM	AGS – 2.5 ± 0.5 g l^‐1^ VSS			Batch anaerobic reactors (25 ml – WV)	VFA (acetic, propionic and butyric acid, 1:10:10) – 2 g l^‐1^ COD	c.a.	0	0	
							0.1	46 ± 5	2.07 ± 0.24	
	o‐NoA – 1 mM	AGS – 2.5 ± 0.5 g l^‐1^ VSS			Batch anaerobic reactors (25 ml – WV)	VFA (acetic, propionic and butyric acid, 1:10:10) – 2 g l^‐1^ COD	c.a.	32 ± 1	0.07 ± 0.01	Pereira *et al*. (2016)
							0.1	97 ± 3	5.28 ± 0.10	
	m‐NoA – 1 mM	AGS – 2.5 ± 0.5 g l^‐1^ VSS			Batch anaerobic reactors (25 ml – WV)	VFA (acetic, propionic and butyric acid, 1:10:10) – 2 g l^‐1^ COD	c.a.	56 ± 4	0.26 ± 0.11	
							0.1	97 ± 1	26.88 ± 0.24	
	p‐NoA – 1 mM	AGS – 2.5 ± 0.5 g l^‐1^ VSS			Batch anaerobic reactors (25 ml – WV)	VFA (acetic, propionic and butyric acid, 1:10:10) – 2 g l^‐1^ COD	c.a.	52 ± 2	0.14 ± 0.02	
							0.1	92 ± 1	23.76 ± 1	
AC _N2_		AS			USPBR (2 ml)	Sodium acetate – 0.2 g l^‐1^	c.a.	n.d.	n.d.	Mezohegyi *et al*. (2010)
	AO 7–0.27 mM						500	>88	8.6^a^	
	Reactive Black 5–0.06 mM						500	>88	8.9 ^a^	
AC _HNO3_	o‐NoA – 1 mM	AGS – 2.5 ± 0.5 g l^‐1^ VSS			Batch anaerobic reactors (25 ml – WV)	VFA (acetic, propionic and butyric acid, 1:10:10) – 2 g l^‐1^ COD	c.a.	32 ± 1	0.07 ± 0.01	Pereira *et al*. (2016)
							0.1	94 ± 1	2.4 ± 0.72	
	m‐NoA – 1 mM	AGS – 2.5 ± 0.5 g l^‐1^ VSS			Batch anaerobic reactors (25 ml – WV)	VFA (acetic, propionic and butyric acid, 1:10:10) – 2 g l^‐1^ COD	c.a.	56 ± 4	0.26 ± 0.11	
							0.1	95 ± 1	5.52 ± 0.24	
	p‐NoA – 1 mM	AGS – 2.5 ± 0.5 g l^‐1^ VSS			Batch anaerobic reactors (25 ml – WV)	VFA (acetic, propionic and butyric acid, 1:10:10) – 2 g l^‐1^ COD	c.a.	56 ± 4	0.26 ± 0.11	
							0.1	94 ± 1	4.32 ± 0.24	
AC _AQS_	CR – 0.22 mM	AGS – 0.1 g l^‐1^ VSS			Batch anaerobic reactors (50 ml – WV)	Glucose – 1 g l^‐1^	c.a.	20 ± 1.8	0.24 ± 0.01	Alvarez *et al*. (2017)
							1.13	67 ± 5.0	1.07 ± 0.07	
	CR – 0.14 mM	AGS – 5 g l^‐1^ VSS			UASB reactor (190 ml – WV)	Glucose – 1 g l^‐1^ COD	9.0	80 ± 2.3	0.54	
						Glucose; *p*‐cresol – 0.6 g l^‐1^ COD	9.0	88± 5.9	0.30	
ACF (KoTHmex)	MR – 0.2 mM.	AGS – 0.5 g l^‐1^ VSS			Batch anaerobic reactors (60 ml – NV)	Glucose – 3 g l^‐1^	c.a.	32	0.237 ± 0.006	Emilia Rios‐Del Toro et al. (2013)
							0.2	92	24.72 ± 0.10	
	4‐Nitrophenol – 0.5 mM	AGS – 25 g l^‐1^ VSS			UASB reactor (400 ml – WV)	Ethanol – 0.025 g l^‐1^	c.a.	38	0.57	Amezquita‐Garcia *et al*. (2016)
							7.2	56	0.84	
ACF _HNO3_	MR – 0.2 mM.	AGS – 0.5 g l^‐1^ VSS			Batch anaerobic reactors	Glucose – 3 g l^‐1^	c.a.	32	0.237 ± 0.006	Emilia Rios‐Del Toro et al. (2013)
							0.2	99	45.12 ± 0.10	
	4‐Nitrophenol – 0.5 mM	AGS – 25 g l^‐1^ VSS			UASB reactor (400 ml – WV)	Ethanol – 0.021 g l^‐1^	c.a.	38	0.57	Amezquita‐Garcia *et al*. (2016)
							8.4	80	1.20	
ACF _AQDS_	4‐Nitrophenol – 0.5 mM	AGS – 25 g l^‐1^ VSS			UASB reactor (400 ml – WV)	Ethanol – 0.021 g l^‐1^	c.a.	38	0.57	Amezquita‐Garcia *et al*. (2016)
							8.4	75	1.13	
BC	Fe (III) – 15 mM	*Shewanella oneidensis* MR‐1–2 × 10^10^ cells ml^‐1^			Anoxic conditions (16 ml tubes)	Lactate – 30 mM	c.a.	< 55	n.d.	Kappler *et al*. (2014)
							10	103 ± 1.5	1.49 ± 0.23^b^	
	Pentachlorophenol – 0.02 mM	Soil bacteria – 25 g l^‐1^			Batch anaerobic reactors (50 ml – SB)	Lactate – 10 mM	c.a.	n.d.	0.011	Tong *et al*. (2014)
							10% (w/w)	100	0.882 ± 0.037	
	Pentachlorophenol – 0.08 mM	*Geobacter* *Sulfurreducens* – 0.9 × 10^10^ cells l^‐1^			Batch anaerobic reactors (50 ml – WV)	Acetate −15mM	c.a.	11.1	220^c^	Yu *et al*. (2015)
							2	85.1	5460^c^	
	Pentachlorophenol – 0.08 mM	*Geobacter* *Sulfurreducens* – 0.9 × 10^10^ cells l^‐1^			Batch anaerobic reactors (50 ml – WV)	Acetate −15mM	c.a.	11.1	220^c^	Yu *et al*. (2015)
BC _AQDS_							2	25.2	810^c^	
BC _Hydroquinone_	Pentachlorophenol – 0.08 mM	*Geobacter* *Sulfurreducens* – 0.9 × 10^10^ cells l^‐1^			Batch anaerobic reactors (50 ml – WV)	Acetate −15mM	c.a.	11.1	220^c^	Yu *et al*. (2015)
							2	34.6	1530^c^	
CXA	MY10 – 1 mM	AGS – 2.5 ± 0.5 g l^‐1^ VSS			Batch anaerobic reactors (25 ml – WV)	VFA (acetic, propionic and butyric acid, 1:10:10) – 2 g l^‐1^ COD	c.a.	83 ± 1	9.50 ± 0.49	Pereira *et al*. (2014)
							0.1	85 ± 1	11.11 ± 0.44	
	RR120 – 1 mM	AGS – 2.5 ± 0.5 g l^‐1^ VSS			Batch anaerobic reactors (25 ml – WV	VFA (acetic, propionic and butyric acid, 1:10:10) – 2 g l^‐1^ COD	c.a.	67 ± 3	3.09 ± 0.30	
							0.1	73 ± 1	3.78 ± 0.19	
	AO10 – 1 mM	AGS – 2.5 ± 0.5 g l^‐1^ VSS			Batch anaerobic reactors (25 ml – WV)	VFA (acetic, propionic and butyric acid, 1:10:10) – 2 g l^‐1^ COD	c.a.	0	0	
							0.1	67 ± 1	2.72 ± 0.13	
	o‐NoA – 1 mM	AGS – 2.5 ± 0.5 g l^‐1^ VSS			Batch anaerobic reactors (25 ml – WV)	VFA (acetic, propionic and butyric acid, 1:10:10) – 2 g l^‐1^ COD	c.a.	32 ± 1	0.07 ± 0.01	Pereira *et al*. (2016)
							0.1	93 ± 2	2.4 ± 0.24	
	m‐NoA – 1 mM	AGS – 2.5 ± 0.5 g l^‐1^ VSS			Batch anaerobic reactors (25 ml – WV)	VFA (acetic, propionic and butyric acid, 1:10:10) – 2 g l^‐1^ COD	c.a.	56 ± 4	0.26 ± 0.11	
							0.1	94 ± 1	5.28 ± 0.72	
	p‐NoA – 1 mM	AGS – 2.5 ± 0.5 g l^‐1^ VSS			Batch anaerobic reactors (25 ml – WV)	VFA (acetic, propionic and butyric acid, 1:10:10) – 2 g l^‐1^ COD	c.a.	52 ± 2	0.14 ± 0.02	
							0.1	93 ± 1	3.36 ± 0.24	
CXB	MY10 – 1 mM	AGS – 2.5 ± 0.5 g l^‐1^ VSS			Batch anaerobic reactors (25 ml – WV)	VFA (acetic, propionic and butyric acid, 1:10:10) – 2 g l^‐1^ COD	c.a.	83 ± 1	9.50 ± 0.49	Pereira *et al*. (2014)
							0.1	85 ± 1	14.99 ± 0.18	
	RR120 – 1 mM	AGS – 2.5 ± 0.5 g l^‐1^ VSS			Batch anaerobic reactors (25 ml – WV)	VFA (acetic, propionic and butyric acid, 1:10:10) – 2 g l^‐1^ COD	c.a.	67 ± 3	3.09 ± 0.30	
							0.1	75 ± 2	4.54 ± 0.67	
	AO10 – 1 mM	AGS – 2.5 ± 0.5 g l^‐1^ VSS			Batch anaerobic reactors (25 ml – WV)	VFA (acetic, propionic and butyric acid, 1:10:10) – 2 g l^‐1^ COD	c.a.	0	0	
							0.1	98 ± 2	4.48 ± 0.75	
	o‐NoA – 1 mM	AGS – 2.5 ± 0.5 g l^‐1^ VSS			Batch anaerobic reactors (25 ml – WV)	VFA (acetic, propionic and butyric acid, 1:10:10) – 2 g l^‐1^ COD	c.a.	32 ± 1	0.07 ± 0.01	Pereira *et al*. (2016)
							0.1	91 ± 1	2.16 ± 0.24	
	m‐NoA – 1 mM	AGS – 2.5 ± 0.5 g l^‐1^ VSS			Batch anaerobic reactors (25 ml – WV)	VFA (acetic, propionic and butyric acid, 1:10:10) – 2 g l^‐1^ COD	c.a.	56 ± 4	0.26 ± 0.11	
							0.1	92 ± 1	8.38 ± 0.24	
	p‐NoA – 1 mM	AGS – 2.5 ± 0.5 g l^‐1^ VSS			Batch anaerobic reactors (25 ml – WV)	VFA (acetic, propionic and butyric acid, 1:10:10) – 2 g l^‐1^ COD	c.a.	52 ± 2	0.14 ± 0.02	
							0.1	91 ± 1	2.4 ± 0.24	
CNT (Nanocyl 3100)	Textile wastewaters 1	AGS – 2.5 ± 0.5 g l^‐1^ VSS			Batch anaerobic reactors (25 ml – WV)	VFA (acetic, propionic and butyric acid, 1:10:10) – 2 g l^‐1^ COD	c.a.	63 ± 2	0.59 ± 0.07	Pereira *et al*. (2014)
							0.1	63 ± 3	0.72 ± 0.07	
	Textile wastewaters 2	AGS – 2.5 ± 0.5 g l^‐1^ VSS			Batch anaerobic reactors (25 ml – WV)	VFA (acetic, propionic and butyric acid, 1:10:10) – 2 g l^‐1^ COD	c.a.	0	0	
							0.1	32 ± 1	6.01 ± 0.69	
	MY10 – 1 mM	AGS – 2.5 ± 0.5 g l^‐1^ VSS			Batch anaerobic reactors (25 ml – WV)	VFA (acetic, propionic and butyric acid, 1:10:10) – 2 g l^‐1^ COD	c.a.	83 ± 1	9.50 ± 0.49	
							0.1	86 ± 1	20.08 ± 1.14	
	RR120 – 1 mM	AGS – 2.5 ± 0.5 g l^‐1^ VSS			Batch anaerobic reactors (25 ml – WV)	VFA (acetic, propionic and butyric acid, 1:10:10) – 2 g l^‐1^ COD	c.a.	67 ± 3	3.09 ± 0.30	
							0.1	75 ± 2	4.01 ± 0.28	
	AO10 – 1 mM	AGS – 2.5 ± 0.5 g l^‐1^ VSS			Batch anaerobic reactors (25 ml – WV)	VFA (acetic, propionic and butyric acid, 1:10:10) – 2 g l^‐1^ COD	c.a.	0	0	
							0.1	98 ± 2	3.16 ± 0.65	
	^14^C‐catechol – 1.3 mM	Soil microbial biomass – 2 g (dry weight).			Batch anaerobic reactors (100 ml – WV)	n.d.	c.a.	18.48 ± 0.85^e^	n.d.	Shan *et al*. (2015)
							0.2^d^	22.00 ± 1.24^e^	n.d.	
	o‐NoA – 1 mM	AGS – 2.5 ± 0.5 g l^‐1^ VSS			Batch anaerobic reactors (25 ml – WV)	VFA (acetic, propionic and butyric acid, 1:10:10) – 2 g l^‐1^ COD	c.a.	32 ± 1	0.07 ± 0.01	Pereira *et al*. (2016)
							0.1	94 ± 6	2.4 ± 0.24	
	m‐NoA – 1 mM	AGS – 2.5 ± 0.5 g l^‐1^ VSS			Batch anaerobic reactors (25 ml – WV)	VFA (acetic, propionic and butyric acid, 1:10:10) – 2 g l^‐1^ COD	c.a.	56 ± 4	0.26 ± 0.11	
							0.1	91 ± 1	2.4 ± 0.24	
	p‐NoA – 1 mM	AGS – 2.5 ± 0.5 g l^‐1^ VSS			Batch anaerobic reactors (25 ml – WV)	VFA (acetic, propionic and butyric acid, 1:10:10) – 2 g l^‐1^ COD	c.a.	52 ± 2	0.14 ± 0.02	
							0.1	91 ± 1	1.68 ± 0.24	
	AO10 – 0.5 mM	AGS – 10 g l^‐1^ VS			UASB reactor (400 ml – WV)	VFA (acetic, propionic and butyric acid, 1:10:10) – 2 g l^‐1^ COD	c.a.	16 ± 4	0.38	Pereira *et al*. (2016)
							1.2	98 ± 3	2.35	
	Textile effluent – 0.5 mM	AGS – 10 g l^‐1^ VS			UASB reactor (400 ml – WV)	VFA (acetic, propionic and butyric acid, 1:10:10) – 2 g l^‐1^ COD	c.a.	31 ± 2	0.37	
							1.2	65 ± 2	0.78	
	AO10 – 0.5 mM	AGS – 2 g l^‐1^ VS			Batch anaerobic reactors (25 ml – WV)	VFA (acetic, propionic and butyric acid, 1:10:10) – 2 g l^‐1^ COD	c.a.	32 ± 0.3	0.27 ± 0.03	Silva *et al*. (2020)
							0.1	97 ± 0.2	2.64 ± 0.16	
	CIP – 0.015 mM	AGS – 3 g l^‐1^ VS			Batch anaerobic reactors (100 ml – WV)	Ethanol – 30 mM	c.a.	95 ± 1.0	1.67 ± 0.4	Silva *et al*. (2021)
							0.1	97 ± 0.7	2.24 ± 0.3	
CNT _HNO3_	Nitrobenzene – 0.8 mM	*Shewanella oneidensis* MR‐1 (OD_600_ = 0.1)			Batch anaerobic reactors (25 ml – WV)	Lactate – 20 mM	c.a.	54	23.6^f^	Yan *et al*. (2014)
							5 g l^‐1^ (5%w/v)	95	39.2 ^f^	
	AO10 – 0.5 mM	AGS – 2 g l^‐1^ VS			Batch anaerobic reactors (25 ml – WV)	VFA (acetic, propionic and butyric acid, 1:10:10) – 2 g l^‐1^ COD	c.a.	32 ± 0.3	0.27 ± 0.03	Silva *et al*. (2020)
							0.1	94 ± 1.2	2.32 ± 0.14	
CNT _N2_	AO10 – 0.5 mM	AGS – 2 g l^‐1^ VS			Batch anaerobic reactors (25 mlL – WV)	VFA (acetic, propionic and butyric acid, 1:10:10) – 2 g l^‐1^ COD	c.a.	32 ± 0.3	0.27 ± 0.03	Silva *et al*. (2020)
							0.1	98 ± 0.1	2.94 ± 0.18	
GO (Graphene Supermarket®)	RR2 – 0.5 mM	AGS – 1 g l^‐1^ VSS		Methanogenic conditions	Batch anaerobic reactors (50 ml – WV)	Lactate/Ethanol – 2 g l^‐1^ COD (0.5:0.5 of COD)	c.a.	50	0.18 ± 0.01	Colunga *et al*. (2015)
							0.005	60	0.36 ± 0.11	
				Sulfate‐reducing conditions – (1g l^‐1^ sulfate)	Batch anaerobic reactors (50 ml – WV)	Lactate/Ethanol – 2 g l^‐1^ COD (0.5:0.5 of COD)	c.a.	n.d.	0.89 ± 0.2	
							0.005	n.d.	3.24 ± 0.10	
	IOP – 0.0005 mM	AGS – 1 g l^‐1^ VSS		Methanogenic conditions	Batch anaerobic reactors (50 ml – WV)	Lactate/Ethanol – 1 g l^‐1^ COD	c.a.	20	12.48 ^c^	Toral‐Sánchez *et al*. (2017)
							0.005	64	34.02 ^c^	
				Sulfate‐reducing conditions – (1g l^‐1^ sulfate)	Batch anaerobic reactors (50 ml – WV)	Lactate/Ethanol – 1 g l^‐1^ COD	c.a.	38	31.2 ^c^	
							0.005	61	61.38 ^c^	
rGO	Nitrobenzene – 1.6 mM	AGS – 1.46 g l^‐1^ VSS			Batch anaerobic reactors (70 ml – WV)	Glucose – 1 g l^‐1^	c.a.	70	n.d.	Wang *et al*. (2014)
							0. 15	>80	n.d.	
	Nitrobenzene – 0.4 mM	Mixture of anaerobic microorganisms – 0.5 g l^‐1^ VSS			Batch anaerobic reactors (100 ml – WV)	Glucose – 1 g l^‐1^	c.a.	n.d.	84.25 ± 2.88^g^	Li *et al*. (2016)
							0.3	n.d.	114.57 ± 1.65 ^g^	
	IOP – 0.0005 mM	AGS – g l^‐1^ VSS	Methanogenic conditions		Batch anaerobic reactors (50 ml – WV)	Lactate/Ethanol – 1 g l^‐1^ COD	c.a.	20	12.48 ^c^	Toral‐Sánchez *et al*. (2017)
							0.005	77	68.76 ^c^	
			Sulfate‐reducing conditions – (1g l^‐1^sulfate)		Batch anaerobic reactors (50 ml – WV)	Lactate/Ethanol – 1 g l^‐1^ COD	c.a.		31.2 ^c^	
							0.005	86	90.31 ^c^	
rGO _N2_	Nitrobenzene – 0.4 mM	Mixture of anaerobic microorganisms – 0.5 g l^‐1^ VSS			Batch anaerobic reactors (100 ml – WV)	Glucose – 1 g l^‐1^	c.a.	n.d.	84.25 ± 2.88 ^g^	Li *et al*. (2016)
							0.3	n.d.	140.31 ± 3.97 ^g^	
Composite nanomaterials										
SBC Zn _400_	AO7 – 0.25 mM	AS – 1.25 g ml^‐1^			UPBR (8 ml – WV)	Sodium acetate	c.a.	n.d.	n.d.	Athalathil *et al*. (2014)
							1250	15	3.6	
SBC Zn _600_								98.3	24.8	
SBC Zn _800_								86	21.7	
SBC Co _600_	AO7 – 0.25 mM	AS – 1.25 g ml^‐1^			UPBR (8 ml – WV)	Sodium acetate – 200 g ml^‐1^	c.a.	n.d.	n.d.	Athalathil *et al*. (2015)
							1250	10	2.5	
SBC Ni _600_								55	13.9	
SBC Fe _600_								57	14.4	
SBC Zn _600_								78	19.7	
FeO	AO10 – 0.5 mM	AGS – 2 g l^‐1^ VSS			Batch anaerobic reactors (25 ml – WV)	VFA (acetic, propionic and butyric acid, 1:10:10) – 2 g l^‐1^ COD	c.a.	31 ± 3	0.21 ± 0.03	Pereira *et al*. (2017)
							1.0	26 ± 6	0.15 ± 0.03	
CoFeO							1.0	31 ± 5	0.19 ± 0.03	
MnFeO							1.0	25 ± 5	0.15 ± 0.03	
C@FeO HdM							1.0	24 ± 4	0.14 ± 0.01	
C@CoFeO CVD750							1.0	87 ± 2	2.69 ± 0.27	
C@CoFeO CVD750._NH3_							1.0	9 ± 3	2.68 ± 0.06	
C@FeO CVD750	AO10 – 0.5 mM	AGS – 2 g l^‐1^ VSS			Batch anaerobic reactors (25 ml – WV)	VFA (acetic, propionic and butyric acid, 1:10:10) – 2 g l^‐1^ COD	1.0	79 ± 1	0.13 ± 2.11	
		Abiotic assay				n.a.		± 439	0.11 ± 0.41	
C@FeO CVD850	AO10 – 0.5 mM	AGS – 2 g l^‐1^ VSS			Batch anaerobic reactors (25 ml – WV)	VFA (acetic, propionic and butyric acid, 1:10:10) – 2 g l^‐1^ COD	1.0	92 ± 1	4.94 ± 0.40	
							0.5	70 ± 1	2.59 ± 0.27	
							0.1	36 ± 8	0.25 ± 0.05	
		Abiotic assay				n.a.	1.0	80 ± 8	3.45 ± 0.20	
							0.5	31 ± 4	3.71 ± 1.60	
							0.1	11 ± 1	0.08 ± 0.01	
C@FeO CVD850 sterile	AO10 – 0.5 mM	AGS – 2 g l^‐1^ VSS			Batch anaerobic reactors (25 ml – WV)	VFA (acetic, propionic and butyric acid, 1:10:10) – 2 g l^‐1^ COD	1.0	67 ± 6	3.75 ± 1.01	
		Abiotic assay				n.a.		62 ± 4	2.87 ± 0.31	
C@FeO CVD750·_NH3_	AO10 – 0.5 mM	AGS – 2 g l^‐1^ VSS			Batch anaerobic reactors (25 ml – WV)	VFA (acetic, propionic and butyric acid, 1:10:10) – 2 g l^‐1^ COD	1.0	93 ± 1	6.15 ± 0.37	
		Abiotic assay				n.a.		94 ± 2	4.70 ± 0.63	
C@MnFeO CVD750	AO10 – 0.5 mM	AGS – 2 g l^‐1^ VSS			Batch anaerobic reactors (25 ml – WV)	VFA (acetic, propionic and butyric acid, 1:10:10) – 2 g l^‐1^ COD	1.0	84 ± 6	3.33 ± 1.39	
		Abiotic assay				n.a.		37 ± 3	0.62 ± 1.49	
C@MnFeO CVD750_·NH3_	AO10 – 0.5 mM	AGS – 2 g l^‐1^ VSS			Batch anaerobic reactors (25 ml – WV)	VFA (acetic, propionic and butyric acid, 1:10:10) – 2 g l^‐1^ COD	1.0	82 ± 7	3.67 ± 0.02	
		Abiotic assay				n.a.		59 ± 11	3.70 ± 0.23	
CNT@2%Fe	AO10 – 0.5 mM	AGS – 2 g l^‐1^ VSS			Batch anaerobic reactors (25 ml – WV)	VFA (acetic, propionic and butyric acid, 1:10:10) – 2 g l^‐1^ COD	1.0	96 ± 1	10.25 ± 1.77	
							0.5	98 ± 3	16.66 ± 2.00	
							0.1	99 ± 1	11.63 ± 0.97	
		Abiotic assay				n.a.	1.0	95 ± 1	13.93 ± 2.94	
							0.5	92 ± 1	13.09 ± 1.10	
							0.1	81 ± 2	11.00 ± 0.53	
	AO10 – 0.5 mM	AGS – 2 g l^‐1^ VSS			Batch anaerobic reactors (25 ml – WV)	VFA (acetic, propionic and butyric acid, 1:10:10) – 2 g l^‐1^ COD	c.a.	32 ± 0.3	0.27 ± 0.03	Silva *et al*. (2020)
							0.1	94 ± 1.4	2.00 ± 0.18	
	CIP – 0.015 mM	AGS – 3 g l^‐1^ VS			Batch anaerobic reactors (100 ml – WV)	Ethanol – 30 mM	c.a.^h^	86 ± 2.2	1.41 ± 0.2	Silva *et al*. (2021)
							0.1 ^h^	88 ± 4.1	1.54 ± 0.3	
CNT@2%Fe _HNO3_	AO10 – 0.5 mM	AGS – 2 g l^‐1^ VSS			Batch anaerobic reactors (25 ml – WV)	VFA (acetic, propionic and butyric acid, 1:10:10) – 2 g l^‐1^ COD	c.a.	32 ± 0.3	0.27 ± 0.03	Silva *et al*. (2020)
							0.1	94 ± 0.6	1.59 ± 0.23	
CNT@2%Fe _N2_	AO10 – 0.5 mM	AGS – 2 g l^‐1^ VSS			Batch anaerobic reactors (25 ml – WV)	VFA (acetic, propionic and butyric acid, 1:10:10) – 2 g l^‐1^ COD	c.a.	32 ± 0.3	0.27 ± 0.03	Silva *et al*. (2020)
							0.1	98 ± 0.1	2.50 ± 0.11	
CNT/AQS/Fe_3_O_4_	Methyl Orange – 0.03 mM	Anaerobic consortia – 2.33 × 10^8^ CFU ml^−1^			Batch anaerobic reactors (100 ml – WV)	Glucose	c.a.	n.d.	n.d.	He *et al*. (2020)
							0.2	97 ± 0.8	n.d.	
	Cr (VI) – 0.2 mM	Anaerobic consortia – 2.33 × 10^8^ CFU ml^−1^			Batch anaerobic reactors (100 ml – WV)	Glucose	c.a.	n.d.	n.d.	
							0.2	98 ± 1.4	n.d.	
CNT/HA/Fe_3_O_4_	Methyl Orange – 0.03 mM	Anaerobic consortia – 2.33 × 10^8^ CFU ml^−1^			Batch anaerobic reactors (100 ml – WV)	Glucose	c.a.	n.d.	n.d.	He *et al*. (2020)
							0.2	80 ± 2.67	n.d.	
	Cr (VI) – 0.2 mM	Anaerobic consortia – 2.33 × 10^8^ CFU ml^−1^			Batch anaerobic reactors (100 ml – WV)	Glucose	c.a.	n.d.	n.d.	
							0.2	82 ± 11.3	n.d.	
rGO/Fe_3_O_4_ nanosacks	IOP – 0.0003 mM	AGS – 10 g l^‐1^ VSS			UASB reactor (330 ml – WV)	Glucose – 1 g l^‐1^ of COD	c.a.	51	0.0003	Toral‐Sánchez *et al*. (2018)
							0.085	82	0.0005	
Fe(OH)_3_@biochar	Quinoline – 0.8 mM	AGS – 6 g l^‐1^ MLSS			Batch anaerobic reactors (250 ml ‐SB)	n.d.	c.a.	75.34	0.30	Li *et al*. (2019); Shi *et al*. (2019)
							1.0	80.22	0.32	
	Pyridine – 1.3 mM	AGS – 6 g l^‐1^ MLSS			Batch anaerobic reactors (250 ml ‐SB)	n.d.	c.a.	10.99	0.07	
							1.0	48.22	0.31	
	Indole – 0.9 mM	AGS – 6 g l^‐1^ MLSS			Batch anaerobic reactors (250 ml ‐SB)	n.d.	c.a.	78.22	0.35	
							1.0	83.22	0.37	
Fe(OH)_3_@PAC	Quinoline – 0.8 mM	AGS – 6 g l^‐1^ MLSS			Batch anaerobic reactors (250 ml ‐SB)	n.d.	c.a.	75.34	0.30	Li *et al*. (2019)
							1.0	90.21	0.36	
	Pyridine – 1.3 mM	AGS – 6 g l^‐1^ MLSS			Batch anaerobic reactors (250 ml ‐SB)	n.d.	c.a.	10.99	0.07	
							1.0	50.23	0.33	
	Indole – 0.9 mM	AGS – 6 g l^‐1^ MLSS			Batch anaerobic reactors (250 ml ‐SB)	n.d.	c.a.	78.22	0.35	
							1.0	85.33	0.38	

AGS, anaerobic granular sludge; AS, anaerobic sludge; c.a., control assay without RM; MLSS, mixed liquor suspended solids; n.a., non‐applicable; n.d., not defined; NV, nominal volume; VS, volatile solids; FS,; flocculent sludge; SB, serum bottles; SS, suspended solids; UAFBR, upflow anaerobic fixed bed reactor; UPBR, upflow packed‐bed reactor; USPBR, upflow stirred packed‐bed reactor; VFA, volatile fatty acid mixture; VSS, volatile suspended solids; WV, working volume.

The units in these studies were ^a^mmol g^‐1^ min^‐1^; ^b^mM h^‐1^; ^c^µg l^‐1^ day^‐1^; ^d^mg kg^‐1^ dry soil; ^e^cumulative release of ^14^CO_2_ from ^14^C‐catechol in soil; ^f^mg l^‐1^ h^‐1^ mg^‐1^ dry weight; ^g^µmol h^‐1^ g^‐1^ VSS; and ^h^results after three reutilizations.

**Fig. 1:**
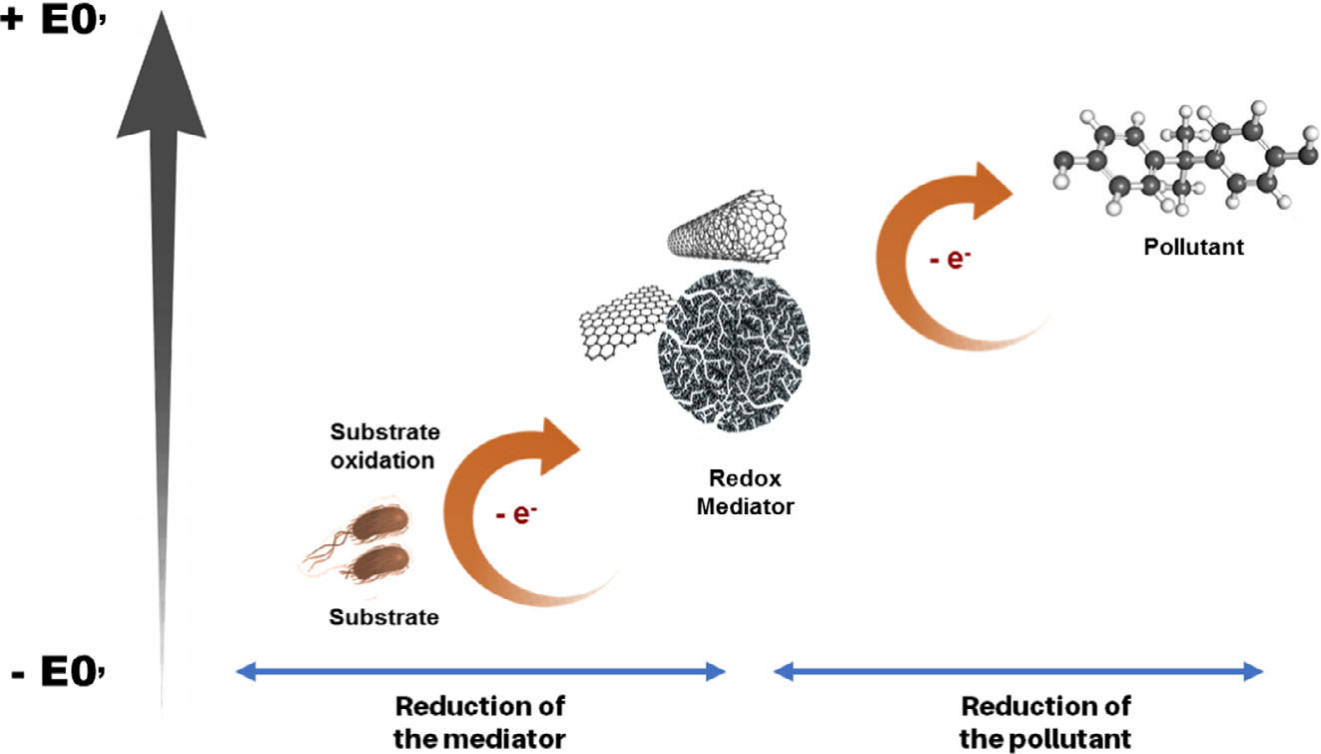
HYPERLINK "sps:id::fig1||locator::gr1" Electron shuttling effectiveness according to the redox potential (E0′) of the system. Ideally, the E0’ is between the two half reactions: the oxidation of a primary electron donor and the reduction of the pollutant.

In the processes catalysed by RM, the reaction begins with the reduction of the mediator by the electrons resulting from the biological oxidation of a substrate, where the reaction rate is favoured when the RM’s E0′ is higher than that of the biological system. Then, in the following step, the reduction of the pollutant by receiving the electrons from the reduced RM is favoured when the E0′ of the RM is lower than that of the pollutant (Fig. 1). Therefore, the balance between these two steps of E0′ is fundamental for the application and the prediction of the ideal RM (dos Santos *et al*., 2007; van der Zee and Cervantes, 2009). Both reaction steps should occur at the same reaction rate. Because reduced and oxidized states alternate, RM can participate in countless reactions, being effective at lower amounts (van der Zee, Bouwman, *et al*., 2001) and acting as a catalyst (van der Zee *et al*., 2003; Cervantes *et al*., 2010; Pereira *et al*., 2010; Xu *et al*., 2013). In the biological processes, the synergetic relation between the CBM, biomass and pollutant is given by the use of CBM as biomass support, adsorbent of pollutants and substrates, and as catalyst of the associated redox reactions, accelerating the biodegradation of the target pollutant (Mezohegyi *et al*., 2012).

Carbon‐based materials can also accelerate the chemical reduction of pollutants, for instance the reduction of azo dyes by sulfide (Pereira *et al*., 2010). Similarly, the reaction starts via chemical reduction of CBM by sulfide, followed by the reduction of the pollutant due to the transfer of electrons from the reduced CBM to it.

Besides, other characteristics of CBM such as having a carbon structure resistant to acidic and basic media, being stable at high temperatures and being able to assume different physical forms, such as granules, pellets, powder and fibres, are also favourable to their application in bioremediation (Rodríguez‐reinoso, 1998; Mezohegyi *et al*., 2012; Dai *et al*., 2016). Also, carbon supports are usually less expensive than other supports, such as silica and alumina. In addition, it is possible to tailor their surface with functional groups for a specific propose, such as targeting to different compounds (Rodríguez‐reinoso, 1998; Toral‐Sánchez *et al*., 2017). On another hand, surface modifications of CBM, either by the incorporation or removal of functional groups, will interfere with the materials S_BET,_ Smeso and microporous volume, as demonstrated by the characterization results presented in Table S1. For instance, the oxidation of CBM with HNO_3_ promotes a slightly reduction of the S_BET_ and of the volume of micropores, due to the collapse of some of the micropore walls caused by the drastic conditions of the treatment and to the incorporation of the functional groups on the carbon structure (Amezquita‐Garcia *et al*., 2013; Emilia Rios‐Del Toro et al., 2013). When applying O_2_ oxidation, textural surface characteristics of AC were not significantly changed; however, the micropore volume, and the average of micropore width, increased (Pereira *et al*., 2010). Regarding thermal treatments with a H_2_ and N_2_ flows, the textural properties of the tailored CBM were nearly maintained, in spite of the decreased of the mesoporous volume by N_2_ treatment.

The increase of mesopores surface area, and of the microporous volume, on these materials, promotes the adsorption of larger molecules, allowing greater mass diffusion and accelerating the reaction kinetics (Gonçalves *et al*., 2010; Pereira *et al*., 2010, 2014; Dai *et al*., 2016; Rocha *et al*., 2017). Furthermore, functional molecules can be incorporated on the surface of CBM, which can be used as extra redox‐active sites (carbonyl structures) (Table 1) (Amezquita‐Garcia *et al*., 2015, 2016; Yu *et al*., 2015; Alvarez *et al*., 2017; He *et al*., 2020). The reduction rates are influenced by other parameters as well: the molecular structure, pKa and redox potential of the compound, and those parameters have also a dependence on the pH of the solution (Pereira *et al*., 2010; Cho *et al*., 2011; Carabineiro *et al*., 2012). The relationship between these variables has been considered as determinant in the efficacy of the biological system for the biodegradation processes.

Other important factor contributing to the efficiency of RM in biological processes is the microbial community involved (dos Santos *et al*., 2006; van der Zee and Cervantes, 2009). During anaerobic digestion, the interspecies electron transfer (IET) between bacteria and archaea communities is crucial for the process (Shen *et al*., 2016). It is noticeable that a stable IET determines the effectiveness of the organic waste’s treatment. Thus, the electron donors (organic or inorganic substrates), when consumed by microbial populations, are progressively reduced into simpler compounds and the final intermediates generated, like hydrogen or formate, will be used by methanogenic communities to produce methane (Batstone and Virdis, 2014; Ghattas *et al*., 2017; Liu *et al*., 2017). Regarding the removal of pollutants, bacteria have been reported to contribute more for the reduction of the pollutant than methanogenic archaea. This occurs, once there is a competition between methanogenesis and the reduction of pollutants for receiving electrons. Methanogens use the reducing equivalents available for methane formation, while acetogenic bacteria assist mediated reactions by promoting the transfer of electrons from the fermented substrate to the pollutants (dos Santos *et al*., 2005; Cervantes and dos Santos, 2011; Wang *et al*., 2014).

Carbon‐based materials have also been described as promoting the direct interspecies electron transfer (DIET), where, contrary to IET, the electron flux occurs directly between bacteria and methanogens, instead of through Hydrogen, so accelerating the rate of CH_4_ production (He *et al*., 2017; Li *et al*., 2017; Yin *et al*., 2017; Rotaru *et al*., 2018). According to some authors, there is the possibility that some conductive CBM may replace cellular structures responsible for the electron shuttling between microbial partners (Lovley, 2017). This hypothesis is based on the fact that in the presence of CBM, instead of using energy to produce biological structures, the cells use the available energy for growth (Liu *et al*., 2012). However, it is not entirely clear whether this occurs, as CBM also enhance the reaction rates in pure cultures of methanogens (Salvador *et al*., 2017). Furthermore, CBM, such as AC, presenting higher size than microbial cells, allow the adhesion of cells on their surface. In this sense, there is no need for microbial partners to be in close physical association, since the connection provided by CBM might be enough (Barua and Dhar, 2017; Lovley, 2017).

### Microporous carbon materials

#### Activated carbon

Several works have showed the high efficiency of AC on the removal of pollutants as absorbent (Oliveira *et al*., 2002; Al‐Degs *et al*., 2008; Ai *et al*., 2010; Mezohegyi *et al*., 2012; Santhosh *et al*., 2016), as catalyst of chemical reactions (Gül *et al*., 2007; Santos *et al*., 2009; Mezohegyi *et al*., 2012; Tahir *et al*., 2020) and also as RM in anaerobic processes for the chemical and biological reduction of a number of pollutants (Mezohegyi *et al*., 2007; Pereira *et al*., 2010; Baêta *et al*., 2013; Pereira et al., 2016a,2016b; Alvarez *et al*., 2017; Lefèvre *et al*., 2018; Li *et al*., 2019; Bonaglia *et al*., 2020; Zhang et al., 2020b). For instance, many studies have demonstrated the effectiveness of AC in the decolourization of dyes, and reduction of aromatic amines, in batch and laboratory‐scale anaerobic reactors (Pereira *et al*., 2010; van der Zee *et al*., 2003; Amezquita‐Garcia *et al*., 2016; Pereira *et al*., 2016), as stated in Table 1. Despite the high adsorption capacity of AC, which facilitates the process, the identification of aromatic amines proved that the removal of azo dyes was mainly due to reduction reactions (van der Zee *et al*., 2003; Gonçalves *et al*., 2010; Pereira *et al*., 2014; Pereira et al., 2016a). In addition, due to the very low amounts of AC commonly used (0.1–1.0 g l^‐1^), the adsorption is negligible when treating high concentrated wastewaters, as is the case of dye containing ones.

The high surface area, chemical structure and functional groups, as well as their availability to be modified physically and chemically (Table 1 and Table S1) aiming at targeting specific contaminants, are important advantages of AC (Pereira *et al*., 2010; Amezquita‐Garcia *et al*., 2013, 2016). The surface chemistry confers to the material an amphoteric character, an important factor to be considered on the catalysis of pollutants (Pereira *et al*., 2010). Based on its amphoteric character, the material may have positively or negatively charged surfaces, depending on the medium pH and on its isoelectric point, commonly represented by the pH at the zero charge point (pH_pzc_) (Pereira *et al*., 2010). Carbon surface becomes negatively charged at pH > pH_pzc_ and positively charged at pH< pH_pzc_. Thus, opposite charges between the pollutant and the CBM will favour the electrostatic interaction and consequently the adsorption/approximation and, consequently, the biotransformation (Órfão *et al*., 2006; Pereira *et al*., 2010, 2017).

For instance, the good performance of AC on the biologic (with anaerobic sludge) reduction of anionic azo dyes was related with its positive surface charge (Pereira *et al*., 2010), since anionic azo dyes, as well as anaerobic sludge, present negative charge in solution at the optimal pH for the microorganism consortia used, pH = 7 (Jia *et al*., 1996; van der Zee *et al*., 2003; Pereira *et al*., 2014).

The functional groups on the surface of AC, i.e. quinone/carbonyl, carboxylic, anhydrides, lactones and phenols (Table S1), are responsible for the AC surface charge and consequent interactions with pollutants and microorganisms. The excellent performance of AC as RM in anaerobic processes is likely because of the high content of delocalized π electrons on the carbon basal planes (Pereira *et al*., 2010), and not just due to the influence of the quinone groups present on AC surface, on the oxidation–reduction reactions, as initially stated (van der Zee *et al*., 2003; Mezohegyi *et al*., 2007). Indeed, the electron‐rich and oxygen‐free sites are responsible for the high catalytic activity and basicity of AC, and of other CBM (Lewis basicity), being also a preponderant characteristic for the reduction of specific compounds (Mezohegyi *et al*., 2010; Pereira *et al*., 2010).

Several strategies can be applied to modify the chemical surface of carbon materials. For instance, starting from a commercial AC, chemical oxidation treatments with nitric acid (HNO_3_), peroxide (H_2_O_2_) and oxygen (O_2_), as well as thermal treatments in hydrogen and nitrogen atmosphere, can be performed, in order to obtain materials with different surface chemical groups, which confer to them acidity or basicity character, without changing significantly their textural properties (Table S1) (Pereira *et al*., 2010; Rivera‐Utrilla *et al*., 2011; Amezquita‐Garcia *et al*., 2013). Liquid and gas oxidation processes are applied for the incorporation of oxygen‐containing groups on the CBM structure (Gonçalves *et al*., 2010; Rocha *et al*., 2017). Through HNO_3_ oxidation treatment, the main functional groups incorporated on CBM surface are carbonyl, carboxyl, anhydrides, lactone and phenol groups, while by the H_2_O_2_ treatment, are the carboxyl, ketone and ether groups (Rivera‐Utrilla *et al*., 2011). The integrated groups on the AC prepared by HNO_3_ oxidation from a commercial AC (AC_HNO3_) were responsible for the high acidity and a decrease of the pH_pzc_ from 8.4 of the original AC to 2.7 (Pereira *et al*., 2010). Phenol and quinone groups were the main groups on AC modified by gas oxidation (AC_O2_), resulting in an AC with a pH_pzc_ of 4.5 (Table S1) (Pereira *et al*., 2010). The oxidation of AC decreased its catalytic efficiency for the biological (with an anaerobic consortia) and chemical (with sulfide) reduction of azo dyes and aromatic amines, in part due to the repulsion of negative AC_HNO3_ and AC_O2_ materials, and the anionic compounds. Moreover, in spite of the higher amount of quinone groups in these oxidized AC, as compared to pristine and thermal treated AC, their effect as RM was surpassed by the large amount of carboxylic acids and anhydrides, which are electron‐withdrawing groups, so hindering the electron transfer. The incorporation of these functional groups by oxidation treatments on ACF and CNT was also reported, but in those studies the catalytic performance of these CNM, for azo dye reduction, benefited from the presence of the quinone groups on CBM surface (Table 1) (Amezquita‐Garcia *et al*., 2013; Emilia Rios‐Del Toro et al., 2013; Yan *et al*., 2014). The involvement of quinone groups on electron shuttle processes is evoked by the work of Liu *et al*. (2012) too, where AC accelerated the electron transfer between *Geobacter metallireducens* and *Geobacter sulfurreducens*, or *Geobacter metallireducens* and *Methanosarcina barkeri*.

Thermal treatments of AC, at high temperatures under N_2_ and H_2_ atmosphere (AC_N2_ and AC_H2_), remove the weakly bounded and highly (re)active carbon atoms like carboxyl, sulfur compounds, nitrogen oxides and carbon dioxide, increasing the polarity and specific interaction with polar compounds, prevailing only a few quinone groups on the AC surface (Rivera‐Utrilla *et al*., 2011; Amezquita‐Garcia *et al*., 2013; Soares *et al*., 2015). So, materials with low oxygen‐containing groups and high basicity can be obtained by the thermal treatment, so exhibiting high values of pH_pzc_.

Thermal treated AC improved its catalytic efficiency for the biological and abiotic reduction of azo dyes (Table 1) which occurred, essentially, due to the ketonic groups remaining on the material surface and the delocalized π electrons of the carbon basal planes, which are more accessible for reductive reactions (Pereira *et al*., 2010; Amezquita‐Garcia *et al*., 2013; Soares *et al*., 2015). Furthermore, the treatment with H_2_ generates a more basic AC, due to the stabilization of the reactive sites by C–H bonds, and enhances the effect of π‐electron system. In N_2_ treatments, some unsaturated carbon atoms are obtained, and the high reactivity of these atoms makes them very susceptible to oxygen adsorption when exposed to air, leading to the reformation of some groups, removed previously during treatment (Pereira *et al*., 2010).

The pH_pzc_ of those modified AC (pH_pzc_ of AC_H2_ = 10.8 and of AC_N2_ = 9.2) is also favourable to the reduction rates due to the opposite charges of materials and dyes, at the pH at which biological reaction was conducted, pH = 7 (Pereira *et al*., 2010). The increase of the first‐order reduction rate constants was more evident in the presence of AC_H2_, essentially due to the more favourable electrostatic interaction with the anionic dyes, consequently facilitating the transfer of electrons. Additionally, the electron density around the azo bond decreased in the presence of electron‐withdrawing groups, such as ‐NH_2_ and ‐OH, easing the reduction, while ‐NH groups are recognized as exerting opposite effect (Pereira *et al*., 2010).

**Fig. 2:**
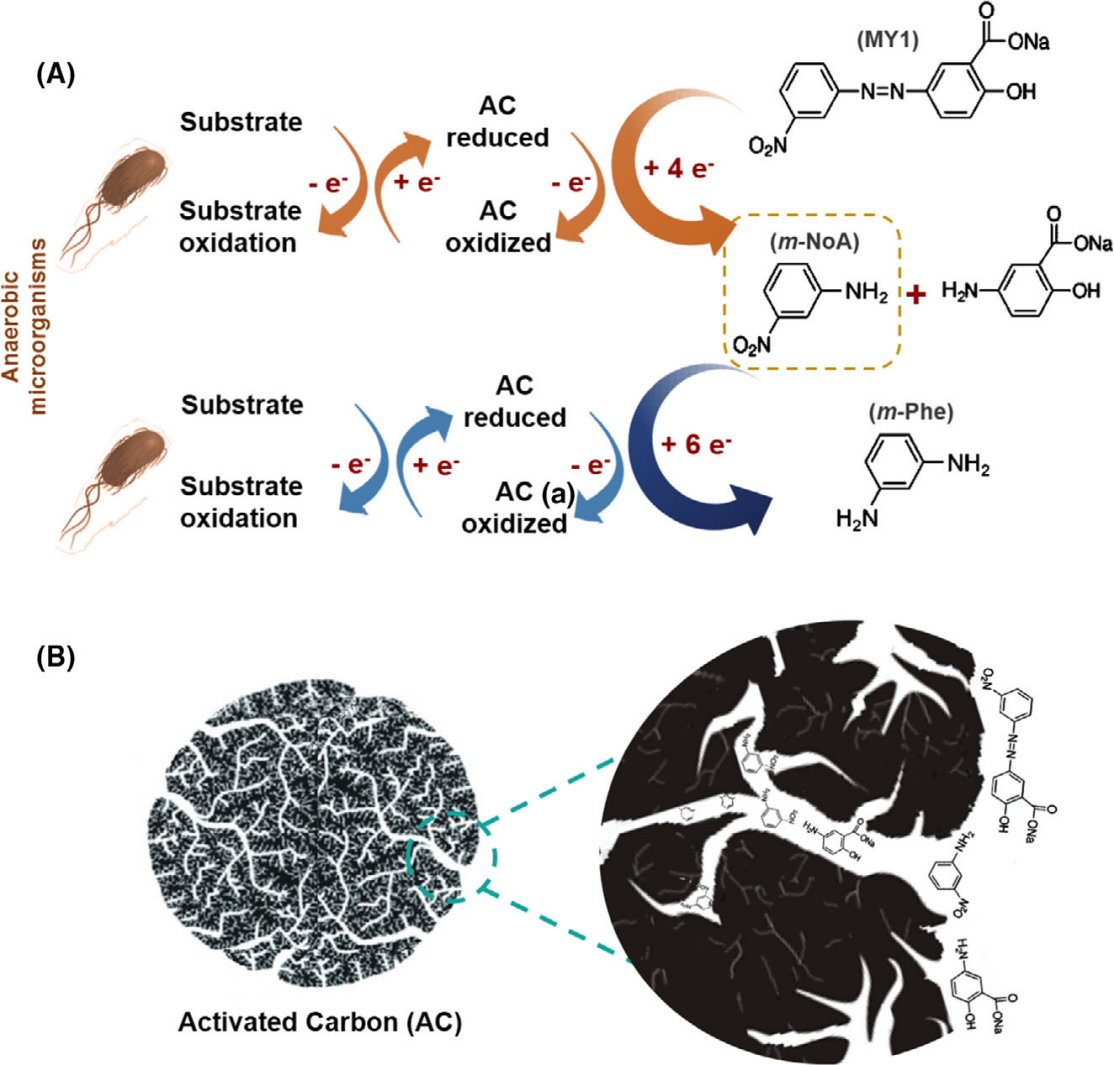
A. Mechanism of anaerobic biodegradation of Mordant Yellow 1 to the corresponding aromatic amines, and further bioreduction of *m*‐NoA to *m*‐Phe, in the presence of AC. B. Schematic representation of MY1 and *m*‐NoA interactions with AC microporous surface: the high molecular size of the dye hinders its entrance in the microporous surface, so the reaction occurs mainly at the surface, while the small molecules of the generated *m*‐NoA can access all the area of the material (outer and inner). Adapted from Pereira et al. (2016a,b).

AC, a material of high porosity, mainly micropores, offers a high S_BET_ which enhance the effectiveness for the uptake of small sized contaminants from aqueous solutions. For instance, Pereira et al. (2016a,b) performed a batch anaerobic reactor set up, testing 0.1 g l^‐1^ of different CBM (AC, CNT and CX) as electron shuttles for the biological removal of nitroanilines (NoA). Despite the removal of nitro aromatic amines under anaerobic conditions being a difficult process due to their nitro groups, creating nitroso and hydroxylamino products through a six‐electron transfer mechanism, and only a few works reporting their degradation (Donlon *et al*., 1997; van der Zee and Villaverde, 2005; Olivares *et al*., 2016), all the CBM tested were excellent RM (Table 1). The higher extents were obtained with AC, up to 98% of NoA removal. These results were explained by the microporous structure and consequent high surface area of AC, promoting a more efficient interaction between the CBM and the pollutants having molecular size sufficiently low to enter the microporous, so making use of all the available contact surface (Pereira et al., 2016a). In other words, the better catalytic performance of AC than of CNT and CX, resulted also from the fact that NoA being small molecules may enter within the porous structure, so the reductive reactions occur not only at the surface of the material but also at inner surface (Fig. 2). Similar to the effect on azo dye reduction, AC_H2_ was more efficient as RM than pristine AC when applied in the reduction of NoA, since NoA are also negatively ionized at neutral pH (Table 1) (Pereira et al., 2016a). In addition, the electron donor substrate, a mixture of volatile fatty acids (VFA), has also negative charge under neutral conditions. Thus, the opposite charges promote electrostatic attraction forces between carbons, VFA and NoA, favouring the electron shuttling process from VFA to CBM and then to NoA (Pereira et al., 2016a). With AC_HNO3_, despite the high removals obtained (up to 95%), the reduction of NoA occurred at lower reaction rates than with AC_H2_ and unmodified AC, because of its negative charge at pH 7 (Pereira *et al*., 2010) resulting in electrostatic repulsion (Pereira et al., 2016a).

It is worth to note that the improvement of the biological reduction of aromatic amines by using AC is very important as aromatic amines resulted from azo dye biotransformation under anaerobic conditions are, in general, recalcitrant to further anaerobic degradation, and can be more toxic than the original dye, so the final treated solution can also present higher toxicity than the dyed wastewater (Pereira, Mondal, et al., 2015).

The removal of diclofenac, ibuprofen, metoprolol, galaxolide and triclosan present in black water was conducted in a 4.7 l upflow anaerobic sludge blanket (UASB) reactor, supplemented with AC. AC promoted less accumulation of those MP, both in liquid phase and in sludge, as well as lower particulate organic matter. Also, only fewer MP were washed out with the effluent and, therefore, authors raise the hypothesis of degradation occurring due to the effect of AC as RM (Butkovskyi *et al*., 2018).

Activated carbon fibres (ACF) have also been proposed as RM of catalytic reactions because they join the advantages of AC as catalysts, and the mechanical strength and flexibility of fibres, this last facilitating their application in bioreactors (Dai *et al*., 2016). As example, Amezquita‐Garcia *et al*. (2016) used ACF as support media for growing anaerobic microorganisms and as RM, in UASB bioreactors, for the biological reduction of 4‐nitrophenol (4NP). The biotransformation of 4NP in the reactor with ACF, above 94%, represented an improvement of 13% in relation to the control reactor (Table 1).

Microorganisms such as *Geobacter*, *Thiobacillus*, *Sulfuricurvum* and methanogenic archaea have been involved in the anaerobic degradation of pollutants in the presence of AC (Zhang *et al*., 2010; Liu *et al*., 2012; Yu *et al*., 2017; Bonaglia *et al*., 2020). Bonaglia *et al*. (2020), investigating the biodegradation of naphthalene, a polycyclic aromatic hydrocarbon (PAH), verified a stimulation of the Deltaproteobacteria genus *Geobacter* in the presence of AC, but not in the absence. Furthermore, *Thiobacillus*, common in soils polluted with PAH (Singleton *et al*., 2013), was found adsorbed on AC. The electrons generated by the oxidation of substrates by *Geobacter* could be transferred via AC between *Geobacter* and *Thiobacillus* facilitating the electrons exchange to PAH (Bonaglia *et al*., 2020). Similarly, the presence of *Sulfuricurvum*, also associated with the degradation of PAH (Zhao *et al*., 2019), was significantly higher in the microbial community in AC supplemented conditions. The same was observed for the methanogenic archaea, *Methanofollis* and *Methanosarcina* (Bonaglia *et al*., 2020). Furthermore, methane production was enhanced in a methanogenic digester in which *Methanosaeta* were the predominant methanogens whose can be stimulated via DIET with *Geobacter* in AC amended cultures (Liu *et al*., 2012).

In the work of Park *et al*. (2018), supplementation of bioreactors with AC promoted a shift in the composition of archaeal community, having reduced the proportion of *Methanosarcina* by 17%, while the proportion of *Methanosaeta* increased by 5.6%. AC also enabled an improvement of the rate and amount of methane production, 72 and 31%, respectively, comparatively to the control without AC (Park *et al*., 2018). Authors have correlated this to changes in composition of the microbial community and to the alterations in the expression of functional genes associated with DIET via AC (Park *et al*., 2018). Conversely, the presence of AC in UASB reactor treating a solution containing Acid Orange 10 (AO10) did not affect the microbial diversity, as no differences were observed between AC‐supplemented and non‐supplemented bioreactor (Pereira et al., 2016b). The most abundant microorganisms in both UASB reactors belonged to the genera *Syntrophobacter*, *Nitrospira*, *Geobacter*, *Pseudomonas*, Syntrophomonas, and 30% to unknown bacteria. Therefore, the effective reduction of AO10 in AC‐bioreactor was attributed to the electron transfer mediated by AC, rather than to changes in the composition of the microbial communities (Pereira et al., 2016b).

#### Biochar

Biochar is a charcoal material obtained by pyrolysis of biomass from raw materials, like wood and bark (Tong *et al*., 2019; Shah *et al*., 2020). It has high specific surface area and contains large amounts of micropores, some mesopores and a small fraction of macropores (Chen *et al*., 2017). The main hindrance for the application of BC is related with its amorphous structure. Diffusion limitations occur when very tight pores are generated, which difficult the uptake of molecules into deeper micropore sites (Tong *et al*., 2019). Despite BC having less porosity and surface area than other CBM, such as AC and CNT (Table S1), it can effectively absorb various organic and inorganic contaminants from soil and water, phenomenon that can be related with BC unique properties such as alkalinity and high ion‐exchange capacity. Furthermore, BC catalytic properties are given by its redox‐active sites, like aromatic and quinones structures (Kappler *et al*., 2014; Tong *et al*., 2014, 2019). The use of BC in the removal of pollutants has been studied, for instance in the biodegradation of pentachlorophenol (PCP) by *Geobacter sulfurreducens* (Yu *et al*., 2015). BC accelerated significantly (>24‐fold) the electron transfer from the microorganism to PCP as a result of its redox‐active moieties and electrical conductivity (Table 1). Furthermore, BC supported the microbial aggregation, working as an inert core, favouring the enrichment on *Geobacter* species. This material also shorted the lag time preceding methanogenesis, by 28.6% (Wang *et al*., 2018).

In another study, the evaluation of the microbial community during the debromination of tetrabromobisphenol revealed that only a small portion (7.2% of the total community relative abundance) of the microbial community responded to BC amendments, so the application of BC did not induce significant changes in the bulk microbial community, being the most representing phyla the Proteobacteria, Bacteroidetes, Actinobacteria, Chloroflexi, Verrucomicrobia, Planctomycetes and Firmicutes (Lefèvre *et al*., 2018). Also, Song *et al*. (2019) observed only a slight effect of BC on the microbial community when this CBM was applied on the removal of petroleum hydrocarbons. However, in the later stage of the reaction *Thiobacillus sp*. abundance increased considerably (Song *et al*., 2019).

Regarding the biological removal of ^14^C‐catechol, the occurrence of *Verrucomicrobia* and *Actinobacteria* was significantly reduced in the presence of BC, while of *Bacteroidetes* increased (Shan *et al*., 2015).

In comparison with AC, a broader fraction of the microbial community was affected by BC (Lefèvre *et al*., 2018). The amorphous structure of BC supports the growth of microbial biofilms and provides a support for nutrients as well, increasing the biomass activity (Inyang and Dickenson, 2015; Frankel *et al*., 2016; Chen *et al*., 2017), while AC, typically having a higher proportion of micropores, is inaccessible to most microbial cells (Pereira *et al*., 2010; Huggins *et al*., 2016). Furthermore, BC might display more redox‐active moieties than AC, hence promoting bacterial growth (Yu *et al*., 2015).

### Macro and mesoporous carbon materials

Macro‐ and mesoporous CBM are reported as new shape catalyst highly efficient for the removal of MP, mainly for larger molecules. This is explained by the easier access of the molecules to the entire surface of the nanocatalyst, avoiding the diffusion limitations of microporous structures (Gonçalves *et al*., 2010; Orge *et al*., 2012; Pereira *et al*., 2014), as outlined before. These carbon nanomaterials (CNM) category include CX, CNT and GNS (Pereira *et al*., 2014; Wang *et al*., 2014; Santhosh *et al*., 2016) and will be covered next.

#### Carbon xerogels

The mesoporous CX provide controlled pore size distribution, high porosity and surface‐active sites, conferring excellent sorption capability and efficiency when compared to granular and powder AC (Santhosh *et al*., 2016). This type of porous structures facilitates the access and diffusion of large molecules to the CNM internal surface, enhancing the electrons transfer to the pollutant and, consequently, its reduction. Table 1 shows the few works on the efficiency of CX when applied as RM in the biological reduction of several pollutants as well as complex effluents.

The texture of the macro‐ and mesoporous catalysts is defined by the geometry of the empty spaces which determines its porosity. The importance of CBM textural characteristics was demonstrated in the work of Pereira *et al*. (2014), where the catalytic performance of mesoporous and microporous CBM was compared in the anaerobic decolourization of Mordant Yellow 10 (MY10), Reactive Red 2 (RR2) and AO10. The dyes were reduced faster in the presence of CX than AC, revelling that the efficiency may be related with the access of the dyes to the internal surface of the macroporous CNM, so facilitating the electron transfer and increasing the reduction rates.

Aiming to access the effect of porous size independently of other characteristics, two CX were synthesized by the sol–gel process at pH 6.25 (CXA) and 5.45 (CXB), resulting in materials with similar surface but different average mesopore diameter (Pereira *et al*., 2014). CXB, presenting higher average of mesopores than CXA (Table S1), showed better efficiency as RM in the biological reduction of all tested azo dyes (Table 1) (Pereira *et al*., 2014).

#### Carbon nanotubes

Similar to CX, CNT are characterized by having high porosity, uniform pore size distribution and surface‐active sites. Due to the macroporous structure of CNT, the biological reduction of large molecules is facilitated, as demonstrated in the work of Pereira et al. (2016a,b) for the reduction of Mordant Yellow 1 (MY1), where higher rates of dye reduction were obtained with CNT than with AC or CX. However, the NoA resulted from MY10 reduction were more effectively reduced in the presence of AC than CX or CNT, due being smaller molecules, as discussed above (Fig. 2.

On another hand, CNT are characterized by high conductivity and low amount of oxygen‐containing surface groups (Table S1), features that promote the better exposure of surface‐active sites, containing high delocalized π electrons, that are easily transferred (Pereira *et al*., 2014; Pereira *et al*., 2016; Silva, Soares, *et al*., 2020). Those characteristics are fundamental for the treatment of complex industrial effluents. The complexity of real effluents can hinder the biological process, due to the possibility of containing components such as salts, detergents, softeners, surfactants and sizing, coating and finishing additives, of which textile effluent is an example (Pereira *et al*., 2014). Despite the complexity of these effluents, the high catalytic activity of CNT was demonstrated in the works of Pereira *et al*., (2014) and Pereira et al. (2016b) (Table 1).

Likewise as AC, the surface of CNT can be tailored to address target pollutants, as for example by the incorporation of N‐groups and oxygen‐rich groups on its structure (Silva, Soares, *et al*., 2020). N‐doped CNT were better RM on the removal of AO10 (Table 1), since doping CNT with heteroatoms (like N) promotes the rearrangement of the electrons in the carbon surface and alters the electronic properties, enhancing their stability and catalytic performance (Figueiredo and Pereira, 2009; Soares *et al*., 2015). The increase of S_BET_ (Table S1) is also beneficial for CNT_N2_ efficiency as catalyst, since there is a greater area of approximation with the pollutant and of exchange of electrons, facilitating the reduction of the dye (Silva, Soares, *et al*., 2020).

As stated previously, the microbial community may suffer alteration according to the pollutant and to the concentration of NM applied on treatment process (Shan *et al*., 2015; Abbasian *et al*., 2016; Zhang et al., 2020a). The presence of CNT was reported to increase the relative abundance of bacterial genera *Bacteroidetes Firmicutes*, *Flavobacteriales*, *Cellulomonas*, *Clostridiales and Pseudomonas*, which are considered to be potential degraders of recalcitrant contaminants in (Wang *et al*., 2008; Xia *et al*., 2010; Shan *et al*., 2015; Abbasian *et al*., 2016).

Single‐walled carbon nanotubes (SWCNT) and multi‐walled carbon nanotubes (MWCNT) have a different effect on the removal of ^14^C‐catechol and on the microbial community in soil (Shan *et al*., 2015) (Table 1). The phylogenetic analysis indicated changes on the microbial community, where Proteobacteria, Actinobacteria, Chloroflexi and Firmicutes were the most dominant groups in both conditions, SWCNT and MWCNT. However, in the treatment with SWCNT at concentrations ranging from 0.2 to 20 mg kg^‐1^, the relative abundances of Verrucomicrobia, *Cyanobacteria* and Gemmatimonadetes were significantly lower than in the control, but the abundances of Bacteroidetes and Elusimicrobia were considerably higher. Contrarily, MWCNT promoted a decrease on the relative abundance of Bacteroidetes, whereas Chloroflexi and Firmicutes have significantly increased. However, no significant difference was observed between MWCNT at > 20 mg kg^‐1^ (Shan *et al*., 2015).

In the treatment of crude oil, the presence of MWCNT induced the increase of the abundance of Acholeplasmatales, Burkholderiales, Chlamydomonadales, Chlorellales, Chromatiales, Desulfovibrionales, Gemmatimonadales and Myxococcales. For instance, *Clostridiales*, *Erysipelotrichales* and *Lactobacillales* quantity augmented with the increase of MWCNT and crude oil concentrations (Abbasian *et al*., 2016).

#### Graphene nanosheets

There is great interest in the use of GNS in several areas, which is due to the fact that these materials are composed of one atom thick sheet, constructed by sp^2^‐bonded carbon atoms, and have a large surface area, a high electrical conductivity and great catalytic activity (Wang *et al*., 2014). The oxygen moieties and the good electrical conductivity of graphene make it interesting also for the accelerating of the electron transfer. GNS may offer advantages in comparison with CNT, since they are single‐layered materials with two basal planes available for pollutant adsorption, while CNT inner walls are not accessible for many pollutants having bigger molecular structures (Santhosh *et al*., 2016). Thereby, graphene oxide (GO) and reduced graphene oxide (rGO) have been applied as RM. GO has higher amount of oxygenated functional groups on its surface, like carboxylic, lactonic, phenolic and carbonyl groups, than other CNM (Table S1) (Colunga *et al*., 2015). rGO, obtained by the removal of the oxidized functional groups on GO, has an electrical conductivity approximately three orders of magnitude higher than that of GO (Wang *et al*., 2014; Li *et al*., 2016).

The positive performance of GO and rGO as RM is also a virtue of their oxidation–reduction potential (ORP), since it is related with the ability of a chemical compound to accept or donate electrons under specific conditions (Toral‐Sánchez *et al*., 2016). The ORP of GO and rGO is about 60 mV and 501.9 mV respectively (Colunga *et al*., 2015; Toral‐Sánchez *et al*., 2016). These differences occur because the carbonyl groups remained after reduction of GO can act as electron acceptors when oxygenated groups are eliminated from the basal plane (Montes‐Morán *et al*., 2004), enhancing the ORP and the ability to accept electrons by rGO (Toral‐Sánchez *et al*., 2016).

GNS were applied as RM in the biological reduction of the azo dye RR2 (Colunga *et al*., 2015), of the pharmaceutical iopromide (IOP) (Toral‐Sánchez *et al*., 2016, 2017) and of nitrobenzene (Wang *et al*., 2014; Li *et al*., 2016). In the work of Colunga *et al*. (2015), the rate of RR2 biological reduction was accelerated by 0.005 g l^‐1^ of GO: twofold, under methanogenic, and 3.6‐fold, under sulfate‐reducing conditions (Table 1). Furthermore, GO presented negative surface charge, while rGO had a positive surface charge. These differences are due to the presence of negatively charged functional oxygenated groups on GO surface. On another hand, when GO is reduced to rGO, the negatively charged functional groups are removed from GO sheets, resulting in an increase of the surface charge (Colunga *et al*., 2015; Toral‐Sánchez *et al*., 2016).

The influence of the surface charge was observed in the work of Toral‐Sánchez *et al*. (2016, 2017) as well, evaluating the effect of GO (pH_pzc_ 2.3) and of rGO (pH_pzc_ 7.25) as RM of the reduction of the halogenated contrast medium IOP. Both accelerated the reduction of IOP, but the performance of rGO was greater to that of GO: removal of IOP with GO was improved 2.7‐fold, under methanogenic, and 1.9‐fold, under sulfate‐reducing conditions, while the rate increase achieved with rGO was 5.5‐fold and 2.8‐fold higher, under methanogenic and sulfate‐reducing conditions respectively (Table 1). According to the authors, deiodination, demethylation, decarboxylation, dehydration and N‐dealkylation were the main multiple reduction reactions on IOP biotransformation (Wang *et al*., 2014; Toral‐Sánchez *et al*., 2017).

rGO has also been used to improve the biotransformation of nitrobenzene (Wang *et al*., 2014; Li *et al*., 2016), and the principal pathway in the anaerobic transformation of nitrobenzene generally includes nitrosobenzene and hydroxylaminobenzene as intermediates, once the decrease of nitrobenzene concentrations was associated with the accumulation of these intermediates. Furthermore, authors demonstrated that the methanogenic community is not involved in the nitrobenzene biotransformation, either in the non‐mediated and mediated reaction with rGO. Instead, the acetogenic community was the principal responsible for promoting the reduction of this compound, as observed when the medium was supplemented with low concentration of vancomycin (0.5 g l^‐1^), a bacteria inhibitor. A decrease of nitrobenzene removal (63%) was observed in comparison with the systems without vancomycin (> 80%) (Wang *et al*., 2014).

The effect of GNS on the microbial community depends on its concentration and the cultivation time (Lefèvre *et al*., 2018; Song *et al*., 2019). Regarding the anaerobic degradation of petroleum hydrocarbons mediated by GO, a slightly influence of this CNM on the microbial community was observed, and the dominant microorganisms were *Paracoccus denitrificans*, *Pseudomonas aeruginosa* and *Hydrogenophaga caeni*. However, for longer periods of cultivation, *Bacillus sp*. appeared in the culture system (Song *et al*., 2019).

### Composite nanomaterials as redox mediators

Various composite NM have been emerging directed towards the application in environmental remediation both as adsorbents and as catalysts (Oliveira *et al*., 2002; Ai *et al*., 2010; Pereira *et al*., 2017). As described in the previous sections, CBM can be easily functionalized for specific applications which opens up new possibilities as RM in the biological removal for a wider variety of pollutants. Nevertheless, although CBM being used at low amounts and being recycled in the same process, their utilization in industrial applications may increase the operational costs (Dai *et al*., 2016). In this sense, it must be possible to recover and reuse them at the end of the processes. Moreover, removing the catalysts from the treated wastewater after the process is required aiming to obtain a clear effluent and avoid possible toxic effects. Magnetic separation is a low cost, simple, quickly and an efficient method of separation. Accordingly, recently new carbon magnetic nanomaterials (C@MNM) have been developed to combine synergistically catalytic and magnetic properties in a composite NM. These C@MNM can be easily retained in bioreactors and recovered at the end of the process by applying a magnetic field, allowing its reuse (Ji *et al*., 2015; Pereira *et al*., 2017; Toral‐Sánchez *et al*., 2018). For instance, Pereira *et al*. (2017) synthetized a set of C@MNM for application as RM on the biological reduction of azo dyes. Core‐shell composites were composed by an inner core of ferrite (FeO and MFeO, M = Mn^2+^ or Co^2+^) coated with a carbon shell by different methods. Contrarily to previous results with single CNM, that reported a significant improvement on the reaction rates with single CNM at concentration of 0.1 g l^‐1^ (Pereira *et al*., 2014), 5–10 times higher amount of C@MNM were required to achieve similar extents of AO10 reduction, as a result of their lower specific area (Table S2). Among the core‐shell materials, the best results were obtained with the composite prepared by carbon vapour deposition (CVD) at 850 °C (C@FeO_CVD850) which improved the AO10 reduction rates up to 23‐, 12‐ and 1.2‐fold, for 1, 0.5 and 0.1 g l^‐1^, respectively, as compared with the rates obtained in the absence of CNM (Table 1).

As for single materials, also for composites the surface chemistry plays a significant role on their efficiency. Similarly, the treatment with NH_3_ promoted a more basic C@MNM material (pH_pzc_ >10), which favoured the electrostatic interaction between the material and the dye, as explained before. In fact, the samples doped with nitrogen (C@FeO CVD750_·_NH_3_) almost duplicated the reduction rate (Pereira *et al*., 2017). Doping CBM with nitrogen atoms also promotes the rearrange of carbon atoms, changing the electron flow, and consequently their electronic and catalytic properties (Figueiredo and Pereira, 2010; Rocha *et al*., 2017). On another hand, materials prepared by the hydrothermal method (C@FeO HdM) had worse efficiency as RM in the removal of the dye, since these materials presented lower amount of carbon and lower pH_pzc_ (6.7) (Pereira *et al*., 2017).

Impregnation of metals on CBM can also bring them other specific surface and chemical properties, that further enhance the catalytic performance of CBM (Athalathil *et al*., 2014; Pereira *et al*., 2017; Toral‐Sánchez *et al*., 2018). An example of this favourable combination is the impregnation of metals, such as zinc (Zn), iron (Fe), nickel (Ni) and cobalt (Co), in sludge‐based carbonaceous (SBC) materials, obtained from the exhausted sludge (Athalathil *et al*., 2014, 2015). Furthermore, the impregnation of metals may increase the catalytic activity, once the metal particles increase the electroaffinities and the electronegativity of the composite. The thermal treatment of SBC increased the composite S_BET_ surface area, pore diameter and total pore volume, explaining the good catalytic reduction of Acid Organ 7 (AO7), as stated in Table S1 and Table S2 (Athalathil *et al*., 2014, 2015). In the work by Pereira *et al*. (2017), however, the impregnation of C@MNM with Co resulted in a worse effect of the material in the anaerobic reduction of AO10.

**Fig. 3:**
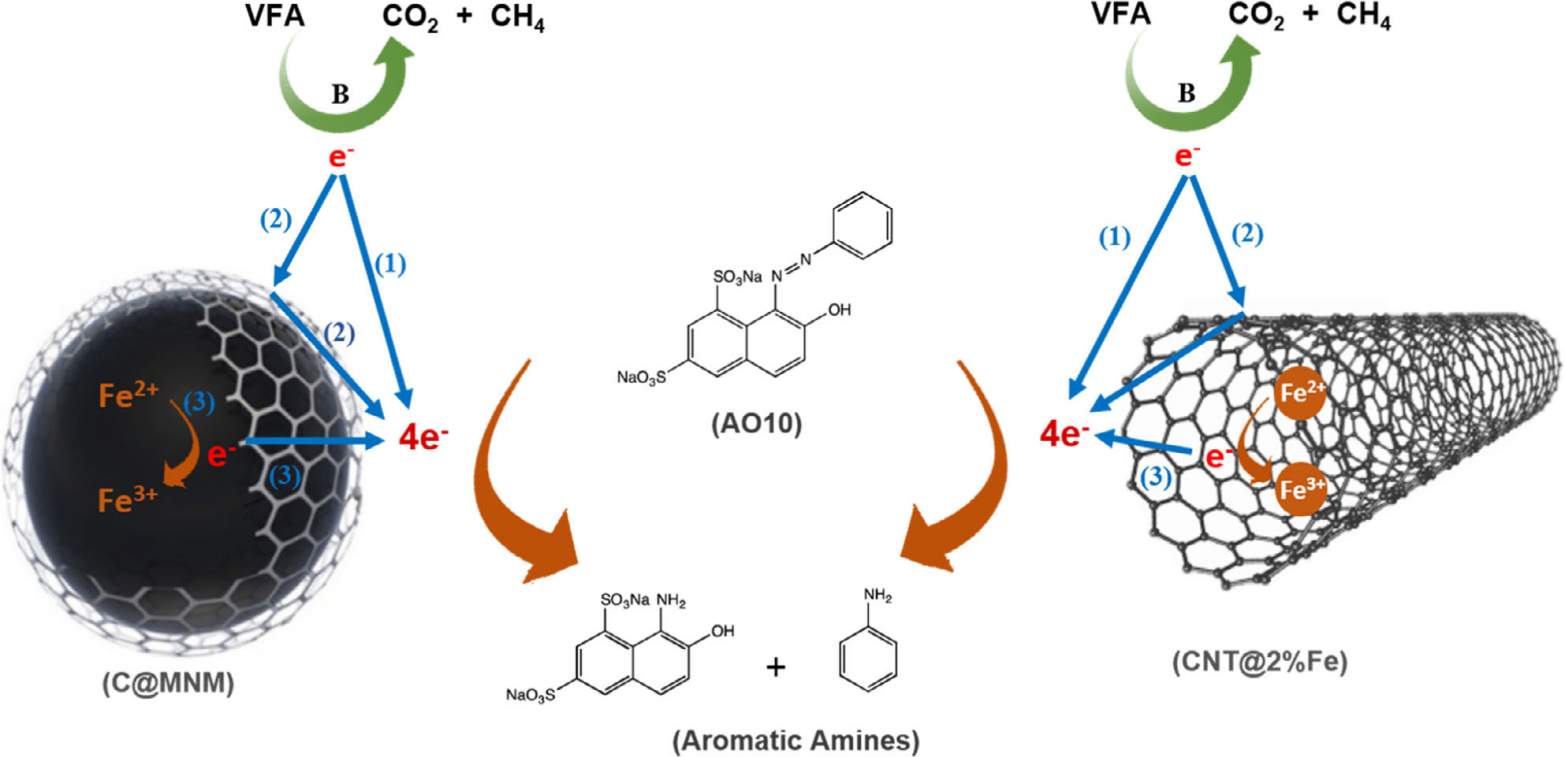
Proposed mechanism of AO10 reduction in the presence of core‐shell C@MNM and CNT@2%Fe composites. Three alternatives of electron flow may be considered: from the biological (B) oxidation of VFA to AO10 (1) or to CM (C@MNM or CNT@2%Fe) composite and then to AO10 (2) and from Fe^2+^ of the core to carbon shell of the composite, or from Fe^2+^ impregnated in CNT composite and then to AO10 (3). Adapted from Pereira et al. (2017).

The incorporation of iron on CNT structure, beyond coffering magnetic properties to the material, increases the catalytic reaction, possibly by the capacity of Fe to transfer electrons to CNT which will further be accessible for the reduction of pollutants (Pereira *et al*., 2014; Ji *et al*., 2015; Toral‐Sánchez *et al*., 2018). Pereira *et al*. (2017) tested a CNT impregnated with 2% of iron as RM in the AO10 anaerobic biological reduction, and a great improvement of AO10 reduction was observed, even at low concentrations of the composite (0.1 g l^‐1^) (Table 1). The nanocomposite accomplished an increasing of 55‐fold, 79‐fold and 66‐fold in the reaction rate, for the concentrations of 0.1 g l^‐1^, 0.5 g l^‐1^ and 1.0 g l^‐1^, respectively, comparatively to the control without CNM. CNT@2%Fe have also showed better performance than single CNT, which denotes that iron participates in the catalysis probably by transferring electrons (Pereira *et al*., 2014, 2017). This was further corroborated by the fact that under abiotic conditions, the reduction of AO10 occurred, as well, only in the presence of this composite NM, probably due to the electron transfer directly to CNT, during iron oxidation (Pereira *et al*., 2017). So, the reduction of the azo dye occurs by the electron flow generated by biological, but also due to abiotic redox mechanisms, explained in Pereira *et al*. (2017). Authors proposed different electron transfer mechanisms that may occur simultaneously (Fig. 3): (i) biological oxidation of the initial electron donor (VFA) to the final electron acceptor, AO10; (ii) electron transfer from the biological oxidation of VFA to carbon material (carbon shell in C@MNM composites or CNT in CNT@2%Fe), and then from the CNM to AO10; and (iii) abiotic degradation of the dye through the oxidation of Fe^2+^ present in the core of C@MNM composites or impregnated in CNT@2%Fe composite, where the electron flows to the carbon of the composites to the final acceptor, AO10 (Pereira *et al*., 2017). Tailored CNT_N2_ (CNT_N_2_@2%Fe) and CNT_HNO3_ (CNT_HNO_3_@2%Fe), where also impregnated with 2% wt Fe in order to compare their performance with that of CNT@2%Fe. The good catalytic accomplishment of the N‐doped CNT and oxidized CNT was maintained after the impregnation with Fe. Accordingly, the improvement of the catalytic activity by doping CNT with nitrogen was also confirmed with CNT_N_2_@2%Fe, where the reduction rate of AO10 increasing 9.3‐fold, coupled to aniline formation at 0.34 mmol L^‐1^ day^‐1^ (Table 1) (Silva, Soares, *et al*., 2020).

CNT@2%Fe was also recently applied on the anaerobic biological removal of the antibiotic ciprofloxacin (CIP). Despite adsorption being negligible in the case of pollutants like azo dyes, owing their high concentrations, for MP, as is the case of pharmaceuticals, absorption accounts for their removal due to the low amounts at which they are usually present. Indeed, different mechanisms for CIP removal, occurring simultaneously, were proposed by Silva *et al*. (2021), including adsorption on anaerobic sludge and on CBM, biological oxidation and biological reduction. Notwithstanding, the contribution of adsorption phenomena was higher in the beginning, until CNT@2%Fe and sludge saturation, but after three degradation cycles of 24 h each, the biological reduction in the presence of CNT@2%Fe seems to be the main removal mechanism (Silva *et al*., 2021).

Tailoring rGO to confer magnetic properties has also been reported (Ji *et al*., 2015; Toral‐Sánchez *et al*., 2018). rGO nanocomposite combining magnetite (Fe_3_O_4_), and silver (Ag) nanoparticles (rGO@Fe_3_O_4_/Ag) was synthetized for the abiotic and chemical degradation of 4NP. The capacity of iron oxide for electron transfer in catalytic reactions was demonstrated by the threefold increase of the reaction rate comparatively to the corresponding rGO/Ag catalyst (Ji *et al*., 2015). Toral‐Sánchez *et al*. (2018) used a magnetic rGO nanosacks (rGO/Fe_3_O_4_ nanosacks) as RM for the bioreduction of IOP in an UASB reactor, adapted with a magnetic trap, in order to retain the magnetic composite within the reactor and easily recover it at the end of the process (Table 1) (Toral‐Sánchez *et al*., 2018). Some by‐products were identified, and the degradation mechanism suggested was similar to the one proposed by Pereira *et al*. (2017).

Another example is the work of He *et al*. (2020), in which magnetic CNT were doped with a quinone (CNT/ Fe_3_O_4_/AQS) and with humic acids (CNT/Fe_3_O_4_/HA), and the good catalytic efficiency demonstrated in the removal of Cr(VI) and of methyl orange was attributed to the combination of the CNT as RM, to the functional molecules, these last acting as redox‐active sites, and still to the electrons generated from the oxidation of Fe^2+^ to Fe^3+^ in the process (Table 1).

Iron oxide (Fe(OH)_3_) incorporated in biochar (Fe(OH)_3_@biochar) and in powder AC (Fe(OH)_3_@PAC) slightly enhanced the removal of nitrogen heterocyclic compounds, compared with single CBM. This was attributed to DIET, the composites acting as electron conductors in the anaerobic system (Li *et al*., 2019; Shi *et al*., 2019). Contrarily to IET based on hydrogen or formate transfer, in DIET no production of intermediates is required, and the electron flux occurs directly between bacteria and methanogens (Li et al., 2015a,2015b; Shen *et al*., 2016; Ghattas *et al*., 2017).

## Toxicity associated with the use of nanomaterials

The extensive synthesis and use of a variety of NM in several areas has not been accompanied by a risk assessment in terms of human health and environmental impact (Cameotra and Dhanjal, 2010; Pereira et al., 2015b; Patil *et al*., 2016; Santhosh *et al*., 2016). Even though nanotechnology being proved as a useful tool for environmental remediation, it is crucial to understand the ecotoxicological effects, mobility, reactivity, mechanisms of action, persistence and bioaccumulation of NM in the environment (Khan *et al*., 2019). One of the major concerns regarding the environmental impact of engineered nanoparticles is that related nanosized particles can enter in water bodies and in drinking water sources, possibly bringing negative consequences to the humans and animals health if continuously exposed to them (Patil *et al*., 2016; Khan *et al*., 2019). When NM are released to water sources, they can be adsorbed on protozoa, bacteria and algae in natural water systems, being transported by these microorganisms to other organisms that feed on them, bioaccumulating and bioamplifying, and thus there is a potential risk to the entire ecosystem and of entering anthropic food chains (Cedervall *et al*., 2012).

Despite that, most of the studies suggesting CBM, and other NM, as RM, do not evaluate the possible risk associated with the discharge of treated water into water resources after treatment. Likewise, knowledge about the effect on the microorganisms exposed to those materials during the treatment biologic process is important to optimize the process and to ensure that the process will not fail during the operation time.

The available studies usually state that CBM and CNM do not cause toxic effects towards anaerobic communities; instead, they increase the methane production rate (Martins *et al*., 2018, 2020; Rotaru *et al*., 2018; Cavaleiro *et al*., 2020). In the studies of toxicity evaluation, the specific methanogenic activity (SMA) has been indirectly related to the potential toxicity of these materials, since methanogens represent the most sensitive microorganisms in the microbial community (Pereira *et al*., 2014; Li *et al*., 2015; Silva, Gomes, *et al*., 2020). However, the short exposition time and low concentration of CBM commonly used in anaerobic treatments may explain, in part, the non‐toxic effect. For instance, in the work of Pereira *et al*. (2014), using AC, CNT and CX at 0.1 g l^‐1^ as RM, none of the CBM caused toxicological impact on the methanogenic community during the 24 h batch experiments. Furthermore, no significant changes on the bacterial and archaeal community were observed in an UASB operating during 77 days in the presence of 1.2 g l^‐1^ AC (Pereira et al., 2016b, Table 2). Notwithstanding, the possibility of accumulation when discharged to the environment must not be discharged, since it may result in amplification of the possible effect, and changes in the microbiome may be expected as well (Pereira *et al*., 2014; Yan *et al*., 2014; Yu *et al*., 2015; Li *et al*., 2016).

**Table 2:** Toxic impact exerted by nanomaterials used in wastewater treatments, towards different microorganisms.

Nanomaterial	Concentration (g l^‐1^)	Microorganism/Inoculum	Toxicity analytical method	Toxic impact	References
AC	0.1	Methanogenic community	Specific Methanogenic activity	n.o.	Pereira *et al*. (2014;. Pereira *et al*. (2016)
CNT	0.005	*E. coli*	Live/dead viability assay (Area‐based estimation)	Loss viability – 24 ± 4%	Kang *et al*. (2008)
	0.005	*E. coli*	Live/dead viability assay/ Plate counting ‐CFUs	Death rate – 59 ± 7%	Liu *et al*. (2009)
	0.08			Death rate – 89 ± 3%	
	1.44	Activated sludge	Respiration inhibition test (sheared sample)	Inhibition – 51 ± 1 %	Luongo and Zhang (2010)
	n.a.	*V. fischeri*	Luminescent assay	EC_50_ – 13.87 mg l^‐1^	Binaeian and Soroushia (2013)
	0.1	Methanogenic community	Specific Methanogenic activity	n.o.	Pereira *et al*. (2014)
	1	Methanogenic community	Specific Methanogenic activity	n.o.	Li *et al*. (2015)
	0.5	Methanogenic community	Specifics methane production rate	n.o.	Cavaleiro *et al*. (2020)
	1				
	0.1	*V. fischeri*	Luminescent assay	Inhibition – 6.8 ± 0.3 % ^a^	Silva *et al*. (2020)
				Inhibition – 4.7 ± 0.7 % ^b^	Silva *et al*. (2021)
CNT _HNO3_	0.1	*V. fischeri*	Luminescent assay	Inhibition – 7.8 ± 2.3 % ^a^	Silva *et al*. (2020)
CNT _N2_	0.1	*V. fischeri*	Luminescent assay	Inhibition – 10.8 ± 5.3 % ^a^	Silva *et al*. (2020)
CNT@2%Fe	0.5	Methanogenic community	Specifics methane production rate	n.o.	Cavaleiro *et al*. (2020)
	0.1	*V. fischeri*	Luminescent assay	Inhibition – 22.3 ± 5.4 % ^a^	Silva *et al*. (2020)
				Inhibition – 18.1 ± 1.7 % ^b^	Silva *et al*. (2021)
CNT@2%Fe _HNO3_	0.1	*V. fischeri*	Luminescent assay	Inhibition – 13 ± 4 % ^a^	Silva *et al*. (2020)
CNT@2%Fe _N2_	0.1	*V. fischeri*	Luminescent assay	Inhibition – 10 ± 2.1 % ^a^	Silva *et al*. (2020)
CNT–Ag nanocomposite	0.05	*E. coli*	Paper‐disc diffusion method	Inhibition zone – 0.9 mm	Dinh *et al*. (2015)
		*S. aureus*		Inhibition zone‐ 0.5 mm	
CX	0.1	Methanogenic community	Specific Methanogenic activity	n.o.	Pereira *et al*. (2014)
GNS					
GO	1	*E. coli*	Colonies counting ‐CFUs	Loss viability – 59 ± 8%	Akhavan and Ghaderi (2010)
		*S. aureus*		Loss viability – 74 ± 5%	
	**0.04**	*E. coli*	Colonies counting ‐CFUs	Loss viability – 69 ± 6%	Liu *et al*. (2011)
rGO	1	*E. coli*	Colonies counting ‐CFUs	Loss viability – 84 ± 3%	Akhavan and Ghaderi (2010)
		*S. aureus*		Loss viability – 95 ± 1%	
	**0.04**	*E. coli*	Colonies counting ‐CFUs	Loss viability – 46 ± 5%	Liu *et al*. (2011)
GO–Ag nanocomposite	n.a.	*P. aeruginosa*	Agar diffusion method	MIC – 2.5 – 5.0 μg ml^‐1^	Faria *et al*. (2014)
	0.05	*E. coli*	Paper‐disc diffusion method	Inhibition zone – 1.5 mm	Dinh *et al*. (2015)
			*S. aureus*		Inhibition zone – 1.0 mm
Nanomaterials in carbon composite materials					
Ag nanoparticles	0.040	Methanogenic community	Specific Methanogenic activity	n.o.	Yang *et al*. (2012)
	0.05	*E. coli*	Paper‐disc diffusion method	Inhibition zone‐ 0.8 mm	Dinh *et al*. (2015)
		*S. aureus*		Inhibition zone‐ 0.5 mm	
nano‐Fe^0^	0.090	*E. coli*	Colonies counting ‐CFUs	Bacteria inactivation (air – saturated) – 2.6 log (log(N/N0)	Lee *et al*. (2008)
Fe_3_O_4_	n.a.	*V. fischeri*	Luminescent assay	IC_50_ – 44.8 mg l^‐1^	Recillas *et al*. (2011)
		*Brachionus rotundiformis*	Mortality from acute exposure	EC_50_ – 722 mg l^‐1^	Mashjoor *et al*. (2018)
Co nanoparticles	n.a.	*Platymonas subcordiforus*	Cell density measurement	EC_50 ‐_ 67.2 mg l^‐1^	Chen *et al*. (2018)
		*Chaetoceros curvisetus*		EC_50 ‐_ 38.6 mg l^‐1^	
		*Skeletonema costatum*		EC_50 ‐_ 21.5 mg l^‐1^	
Ni nanoparticles	n.a.	Zebrafish *Danio rerio* larvae	Mortality from acute exposure (96 h)	LC_50_^5^ = 122.2 mg l^‐1^	Boran and Şaffak (2018)
Zn nanoparticles	n.a.	*Artemia salina*	Mortality from acute exposure (96 h)	LC_50_ ~ 100 mg l^‐1^	Ates *et al*. (2013)

CFUs, colony forming unit; EC_50_, concentrations of compound reducing the bioluminescence by 50% (mg l^‐1^); IC_50_, half maximal inhibitory concentration (mg l^‐1^); LC_50_ – concentrations of compound which cause 50% of organism’s death (mg l^‐1^); MIC, minimum inhibitory concentration; n.a., non‐applicable; n.o, no observed effects.

^a^ Toxicity analysis of the anaerobic medium after incubation with 0.1 g l^‐1^ of CNM for 48 h.

^b^ Toxicity analysis of the anaerobic medium after incubation with 0.1 g l^‐1^ of CNM for 72 h.

On the another hand, the toxicity of CBM and NM is dependent on the organisms used as test agents and on the physico‐chemical properties of the material itself (Pasquini *et al*., 2012; Pereira *et al*., 2014; Santhosh *et al*., 2016). The test organisms traditionally used in bioassays can be grouped into microalgae and plants, fish, crustaceans, rotifers and microorganisms (Fernández‐Alba *et al*., 2002; Mendonça *et al*., 2009; Rizzo, 2011). These toxicological bioassays differ essentially on the exposition time, sensibility of the organisms and reproducibility of the bioassay, and the use of more than one may make sense to better gauge the effect (Persoone and Dive, 1978; Hassan *et al*., 2016).

The choice of the agent will also dependent on what is to be assessed: acute or chronic toxicity (Vosylienė, 2007). Usually for acute toxicity assessment, the choice of bacteria or crustaceans may be more adequate, since these organism present high sensitivity at short exposition times assays (Bird, 2001; Akhavan and Ghaderi, 2010; Rizzo, 2011; Ates *et al*., 2013; Vasquez *et al*., 2013). On the other hand, for chronic toxicity assessment or real‐time analysis, microalgae or fish bioassays may be more indicated, since the exposition time is longer. However, the toxicological methods using these organisms have the disadvantage of being difficult to standardize and to reproduce (Bitton and Koopman, 1992; Minetto *et al*., 2014; Boran and Şaffak, 2018; Xue *et al*., 2018).

The selection of the biological agent used for the toxicity assessment must be done carefully, since different organisms can experience dissimilar toxic effect, which can lead to unlike interpretations. It is necessary to know the context in which CBM and CNM will be applied, to extrapolate the potential toxic effect of the system, as demonstrated on various studies summarized in Table 2. An example of these differences was observed in the work of Recillas *et al*. (2011), which reported that magnetite (Fe_3_O_4_) impute toxic effect towards *V. fischeri* – half maximal inhibitory concentration (IC_50_) of 44.8 mg l^‐1^, after 15 min of incubation time, so being considered as moderately toxic. However, these nanoparticles are considered low toxic towards the *Brachionus rotundiformis* rotifer – half maximal effective concentration (EC_50_) of 722 mg l^‐1^, after 48 h of contact (Mashjoor *et al*., 2018).

Nanomaterials intrinsic properties, such as particle shape and size, specific surface area, hydrophobicity, chemical composition and redox potential, as well as extrinsic properties, including agglomeration rate and surface affinity, dissolution rate and solubility, are important factors possible contributing for their noxious effects (Gatoo *et al*., 2014; Gao and Lowry, 2018). The physical properties of CNM and their interaction with cells seem to be the main mechanism for toxicity induction in microorganisms, instead of oxidative stress as previously stated (Luongo and Zhang, 2010; Pasquini *et al*., 2012).

CNT possess sharp edges which can interfere with the bacterial membrane, acting as ‘nano darts’ and consequently causing the cell death (Kang *et al*., 2008; Liu *et al*., 2009; Binaeian and Soroushia, 2013). Thus, the exposure of microorganisms to SWCNT (at 100, 200, 500 μg g^−1^ soil) and to MWCNT (at 100, 500, 1000 μg g^−1^ soil) exerted toxic effects on the microbial biomass, but MWCNT causing minor effects than SWCNT (Chen *et al*., 2015; Shan *et al*., 2015; Zhang et al., 2020a). The surface physical characteristics of graphite (Gt), GO and rGO, also have been stated as the main causes of toxicity induction towards anaerobic microorganisms (Akhavan and Ghaderi, 2010; Liu *et al*., 2011; Bianco, 2013). These nanosheeted CNM have demonstrated cytotoxic effects towards gram‐positive and gram‐negative bacteria, due to their superior charge transfer, that increased the direct contact between cells and their extremely sharp edges, imputing a membrane stress (Akhavan and Ghaderi, 2010; Liu *et al*., 2011; Wang *et al*., 2014). Liu *et al*. (2011) studied the effect of GO and rGO towards *Escherichia coli (E. coli)*, and a strong toxic effect was observed, with loss of *E*. *coli* viability of (69.3 ± 6.1)% and (45.9 ± 4.8)%, respectively, by 40 µg ml^‐1^ of the NM (Table 2). The particle size and aggregation of graphene nanosheets plays an important role in the antibacterial mechanism of these GNS. In that study, rGO dispersion mainly contained large aggregated particles (2.75 ± 1.18 µm), while GO presented smaller size (0.31 ± 0.2 µm), increasing their interaction with the cells and consequently the higher toxic effect. Increasing the exposition time, and duplicating the GNS concentration, further increased the effect (Liu *et al*., 2011). Opposite results were observed by Akhavan and Ghaderi (2010), i.e. the toxic effect caused by rGO nanowalls was 2.6‐fold higher than that of GO nanowalls, as assessed by *E*. *coli*, and 5.2‐fold for *S. aureus* (Akhavan and Ghaderi, 2010). The higher toxicity of rGO was attributed to the sharper edges of the rGO’s nanowalls, nearly unprovided of functional groups, which leads to a stronger interaction between the bacteria and the nanowalls (Akhavan and Ghaderi, 2010).

It is also worth to mention that surface chemistry also may influence the toxicity of CNM. GO have high density of functional groups on its surface and these surface groups hinder the direct contact of GO nanowalls with the cell membrane, revealing less toxicity when compared with rGO (Akhavan and Ghaderi, 2010). Similarly, functionalized CNT_HNO3_ have demonstrated less toxicity that raw CNT, since the introduction of carboxylic and hydroxyl groups on the tips and sidewalls of CNT_HNO3_ hampers the microorganisms to reach the CNT structure (Kang *et al*., 2008; Liu *et al*., 2009; Pasquini *et al*., 2012).

Silva, Soares *et al*. (2020) evaluated the potential toxic effect that CNM may infer to the treated medium in anaerobic biodegradation assays, by applying the standard *Vibrio fischeri* assay, where the decrease of luminescence inhibition is related with toxic effects. The medium incubated for 24 h with CNT, CNT_HNO3_ and CNT_N2_, at concentration of 0.1 g l^‐1^, did not infer toxic effects towards *Vibrio fischeri*, being the luminescence inhibition extent obtained considered negligible (Table 2).

Assessing the toxic impact of composite NM that combine carbon and metals is also relevant because the metals applied, such as iron, zinc, silver and cobalt, are reported to be toxic for several microorganisms, even at low concentrations (Demirel, 2016; Pereira *et al*., 2017). Metallic oxide nanoparticles enter in WWTP and their impact on biological waste treatment systems have been investigated (Demirel, 2016). Though iron not being considered hazardous, its effect at nanoscale for humans or other organisms is still uncertain. Nanoscaled zero valent iron (nano‐Fe^0^) was efficient on dye decolourization by a microbial culture; however when in concentrations above 4 mg l^‐1^, the microbial activity was compromised (Adebiyi *et al*., 2011). Other studies have described nano‐Fe^0^ as a toxic compound, creating oxidative stress on microorganisms, damaging their membranes and eventually leading to cells death (Lee *et al*., 2008; Dong *et al*., 2019). The toxic mechanisms provided by this element are related with the iron oxides (reduced iron species, Fe^2+^ and/or Fe^0^) which can enter the cells, generating reactive oxygen species (ROS), or from the disturbance of electronic and ionic transport chains of the cell, due to the strong affinity of nanoparticles to the cell membranes (Auffan *et al*., 2008; Lee *et al*., 2008). Furthermore, cell membrane disruption when *E*. *coli* was exposed to nano‐Fe^0^ was observed as result of the reaction of Fe^2+^ with intracellular oxygen or hydrogen peroxide (Table 2) (Lee *et al*., 2008).

The presence of 2 % wt Fe in the composite CNT@2%Fe may also contribute for the final toxicity of the treated effluent. CNT at concentration of 0.1 g l^‐1^ did not infer toxic effects to the anaerobic medium, whereas the composite CNT@2%Fe caused about 20 % of luminescence inhibition towards *V. fischeri* (Silva, Soares, *et al*., 2020; Silva *et al*., 2021). The toxic effect observed is attributed to the Fe in the composite, which can be leached from the CNT during the incubation time, and due to the high affinity of iron oxides to the cell membranes, generating ROS, which could lead to cells death (Table 2) (Silva, Soares, *et al*., 2020; Silva *et al*., 2021). However, despite the possible contribution of CNT@2%Fe to the toxicity of the final treated solution, a 46% of detoxification was obtained after the biological treatment of a CIP solution catalysed by CNT@2%Fe (Silva *et al*., 2021).

According to the study of Faria *et al*. (2014), evaluating the toxicological impact of GO‐Ag nanocomposite, as bactericidal, the toxic effect of Ag on *P. aeruginosa* was stronger than that of GO (Faria *et al*., 2014). Furthermore, the GO‐Ag nanocomposite infers greater bactericidal effect than the single GO, indicating a potentiation of the toxic effect by Ag (Table 2). This toxicity was attributed to the synergy of membrane stress, mediated by the direct physical interactions between GO–Ag composite and the cell membranes, and to the oxidative stress, caused by the induction of ROS mediated by the GO and Ag NM (Dinh *et al*., 2015). In another case, the inhibitory effect of a CNT‐Ag nanocomposite was similar for the single Ag, indicating that the toxic effect is given essentially by the toxicity of Ag nanoparticles (Dinh *et al*., 2015).

Other metals like Co, Ni, Zn and Fe have been impregnated in CBM, e.g. on CNT and on SBC, to enhance its catalytic properties, as mentioned above. The limitation of using these metals may be related to their individual toxicological so possibly also potentiating the toxicological effect of the composite. For instance, the low efficiency of the composite combining SCB and Co was related with the toxicity exerted on the anaerobic culture by Co, co‐inhibiting microbial growth (Athalathil *et al*., 2015). The toxicity of cobalt nanoparticles, as observed for algae, is associated with the cation Co^2+^, when released to the medium (Chen *et al*., 2018).

Regarding Ni nanoparticles, they expressed less acute toxicity in zebrafish *Danio rerio* larvae, than its ionic form Ni^2+^ (Table 2). Despite that, Ni nanoparticles induced alteration on the gene expression, which is primarily associated with the release of Ni ions, promoting oxidative stress (Boran and Şaffak, 2018).

The toxicological impact of Zn nanoparticles was tested on *Artemia salina* larvae. Although no significant acute toxicity was observed in 24 h of exposure, after 96 h the mortality increased significantly, 42%, reflecting a LC_50_ around 100 mg l^‐1^ for Zn nanoparticles with size ranging 40–60 nm (Table 2). This toxic effect is attributed to oxidative stress induced by zinc ionic species (Zn^2+^) released to the medium from Zn nanoparticles (Heinlaan *et al*., 2008; Silva *et al*., 2016). Furthermore, smaller nanoparticles (40–60 nm) caused higher toxicity than larger nanoparticles (80‐100 nm), indicating a size‐dependent toxicity of Zn nanoparticles (Ates *et al*., 2013).

## Concluding remarks and future perspectives

Nanotechnology is expanding in novel environmental technologies for site remediation and wastewater treatment, focusing on synthesis of new materials and improvement of existing materials. The development of novel nanoscale materials, and processes, for treatment of surface and groundwater, and soils, contaminated with organic and inorganic substances, such as industrial chemicals, pesticides and pharmaceuticals, would be the major environmental contribution of nanotechnology.

Wastewater treatment plants represent a primary barrier against the spreading of various pollutants (Grandclément *et al*., 2017; Krzeminski *et al*., 2019). However, the conventional WWTP are not designed for the removal of MP (Luo *et al*., 2014; Bui *et al*., 2016; Dong *et al*., 2016; Rizzo *et al*., 2019). In alternative, anaerobic bioprocesses, i.e. anaerobic digestion, have been proposed. Anaerobic digestion is a very attractive process, but for the biotransformation of those recalcitrant compounds, requires long sludge retention times (SRT) and HRT so that reactions can occur (Stasinakis, 2012; Pereira et al., 2016b; Dubey *et al*., 2021). The prolongation of the exposure times allows the increase of microbial diversity and the retention of slow‐growing organisms, potentiating the biodegradation of pollutants. However, this could represent a drawback for its application in high‐rate anaerobic bioreactors (van der Zee *et al*., 2003; Ju and Zhang, 2015; Harb *et al*., 2016; Pereira *et al*., 2016). The incorporation of insoluble CNM into bioreactors has a potential to improve the removal of pollutants and the overall reaction rates (Table 1), and without the need of being added continuously. This innovative approach is based on the unique physical and chemical properties of those materials that make them valuable for environmental biotechnological applications with the possibility to overcome the weakness of conventional technologies. The combination of the physico‐chemical properties of these NM coupled with their high conductivity provides them the ideal conditions to be applied as RM, accelerating the rated of reaction to realistic values that are compatible with reactors operation. Additionally, the possibility of modifying and tailoring their surface aiming at targeting specific pollutants makes them very attractive to be used in several contexts. There are also evidences that CNM can induce changes in the microbial abundances in contaminated sediments, a fundamental aspect for bioremediation.

In the case of micropollutants (MP), the fact of being present in effluents at very low concentrations, ranging from μg l^‐1^ to ng l^‐1^, is also considered a limitation for their removal in WWTP, in addition to their recalcitrant nature. MP concentrations are orders of magnitude less than other carbon sources typically found in domestic wastewater, thus not being the primary carbon source for the microorganisms, neither the primary electron acceptor. So, in a real context, for wastewater treatment, anaerobic digestion catalysed by CNM could be applied as a tertiary treatment, at which stage the nutrients that are in excess have been removed. Anyway, although the many studies of NM and CBM as RM concern on wastewater treatment, a potential application of anaerobic digestion mediated by CNM would be on the digestion of sludge were the MP adsorb, so being concentrated. For instance, sewage sludge from different sites in the United States contained pharmaceuticals at concentrations up to 11 900 μg Kg^‐1^ of dry‐weight sludge (US EPA, 2009). The application of CNM for the removal of MP on sludge will also allow the valorization of the sewage sludge through the upgrading of the anaerobic digestion process usually applied in WWTP, yielding a nutrient‐rich digestate that may later be used as a soil fertilizer. In addition, it will enable the production of a renewable energy (biogas). The organic matter of sludge will serve as substrate for microorganisms, so an additional source is not required (it is worth to mention that in the most of the research studies on wastewater treatment with RM, a carbon source is added to bioreactors for the generation of electrons).

The treatment of real wastewaters is challenging since, despite the MP, there are also other compounds, as for example concentrated salts, that may interfere with the process and probably need to be removed or diluted before the anaerobic process. However, the few works with real effluents, for instance the improvement of the decolourization of industrial textile effluents in batch, and UASB bioreactors, by applying AC and CNT (Pereira *et al*., 2016), demonstrated the possibility of applying CBM in anaerobic bioprocesses for the degradation of recalcitrant pollutants not only in municipal WWTP, where compounds like salts and detergents are already diluted, but also in treatment plants at industries (for water recycling and/or discharge to the municipal WWTP, respecting the legal requirements). Removal of contaminants from wastewater and recycling of the treated water would provide significant reductions in cost for the industries and increase their eco‐friendliness. So, the use of NM and CBM in the wastewater treatment processes has a potential to accelerate and, in many cases, to allow reactions to occur, allowing to respond mainly to industries which generate large amounts of contaminated effluents with toxic and non‐biodegradable compounds.

The upscale of anaerobic technologies requires a personalized study case by case, since the biodegradation pathways involved on pollutant biodegradation may include several sequential and parallel reactions (Pereira *et al*., 2014; Silva *et al*., 2021). Besides that, the operational conditions, such as HRT, SRT, organic loading rate (OLR) and CNM concentration, also contribute to the effectiveness of the treatment and must be carefully studied. Thus, performing the studies in batch reactors, of little volume, is important to study the parameters and optimize the process as well as choosing the best CNM for a further upscale. Nevertheless, some studies have been emerging on the application of CNM in anaerobic continuous bioreactors, as well as studies on the upscale of these technologies (Amezquita‐Garcia *et al*., 2016; Pereira *et al*., 2016; Alvarez *et al*., 2017; Butkovskyi *et al*., 2018; Toral‐Sánchez *et al*., 2018). Although the volumes being still far from the reality, the good results obtained in these up‐scaled studies demonstrate a great potential for application on even larger scales. As example, the excellent results of the anaerobic biodegradation of the recalcitrant dye AO10, in batch reactors (25 ml) amended with CNM (Pereira *et al*., 2014), were also achieved in continuous bioreactors (400 ml) operating at an HRT of 5 h (Pereira *et al*., 2016). This demonstrates that up‐scaling the process 16 times did not compromise the efficiency of CNM's performance. Other example is the recent study on the application of a magnetic nanomaterial (MNM) (98% metals basis, particle size ranged 50–300 nm) as RM of the decolourization of sulfonated azo dyes. It was conducted in a batch reactor (120 ml) and then up‐scaled to a 4.7 l continuous‐flow UASB reactor (39 times scaling‐up) (Qin *et al*., 2021). The addition of MNM improved the extent of azo dye removal, as well as the anaerobic system resistance to environmental stress, and accelerated sludge granulation. Notwithstanding, the applicability of CNM in environmental bioremediation is dependent on the development of effective technologies to retain them in the reactors or in contaminated sites, and later to separate and remove them, after the treatment. New carbon composite magnetic nanomaterials (C@MNM) are emerging in order to address this issue, combining catalytic and magnetic properties in a composite NM. Thus, C@MNM are promising catalysts to be applied in real contexts. Finally, potential risks, side‐effect and safety aspects have been discussed. A number of studies have emerged assessing the toxicity of NM, using several biological agents. However, the toxic impact of the use of these NM must be studied case by case, according to the specific application. So, further research on the impact of using those potent catalysts is crucial, as well as understanding the mechanisms and factors responsible for toxicity, and risk management tools are of paramount importance in the field of nanotechnology for environmental remediation applications. This knowledge may assist for creating efficient catalysts with low impact, or ways of retaining them in the process and removing after the treatment.

## Funding information

This study was supported by the Portuguese Foundation for Science and Technology (FCT) under the scope of the strategic funding of UID/BIO/04469/2019 unit and BioTecNorte operation (NORTE‐01‐0145‐FEDER‐000004) funded by the European Regional Development Fund under the scope of Norte2020 – Programa Operacional Regional do Norte. Ana Rita Silva holds an FCT grant SFRH/BD/131905/2017.

## Conflict of interest

None declared.

## Supporting information


**Table S1.** Surface modifications and characterization of carbon nanomaterials.
**Table S2.** Tailored nanomaterials used as redox mediators in anaerobic remediation of pollutants: method of preparation and results of characterization.
