# Supplementary material for: Progress and prospects of applying carbon‐based materials (and nanomaterials) to accelerate anaerobic bioprocesses for the removal of micropollutants
Source: Microb Biotechnol. 2021 Sep 29;15(4):1073–100. doi: 10.1111/1751-7915.13822 (PMC8966012; doi:10.1111/1751-7915.13822)
Supplement: Supplementary file 1 — Table S1. Surface modifications and characterization of carbon nanomaterials. Table S2. Tailored nanomaterials used as redox mediators in anaerobic remediation of pollutants: method of preparation and results of characterization. [file MBT2-15-1073-s001.docx]

**Supplementary Information**

**Table S1. Surface modifications and characterization of carbon nanomaterials**

| **Redox mediator** | **Preparation/Modification** | **S_BET_**  **(m^2^ g^-1^)** | **Smeso**  **(m^2^ g^-1^)** | **Micropore volume (cm^3^ g^-1^)** | **Quinones/ carbonyl**  **groups**  **(µmol g^-1^)** | **Carboxylic acids**  **(µmol g^-1^)** | **Anhydrides**  **(µmol g^-1^)** | **Lactones**  **(µmol g^-1^)** | **Phenols (µmol g^-1^)** | **pH_pzc_** | **References** |
| --- | --- | --- | --- | --- | --- | --- | --- | --- | --- | --- | --- |
| **AC** | Commercial Norit Rox 0.8 | 1032 | 138 | 0.382 | 307 | 110 | 79 | 54 | 428 | 8.4 | (Pereira *et al.*, 2010) |
|  | Commercial Norit C Gran | 1400 | n.d. | n.d. | n.d. | n.d. | n.d. | n.d. | n.d. | 4.2 | (Butkovskyi *et al.*, 2018) |
| **AC _HNO3_** | Chemical oxidation with 6M of HNO_3_ at boiling temperature, 3 h | 893 | 102 | 0.346 | 1232 | 723 | 222 | 158 | 948 | 2.7 | (Pereira *et al.*, 2010) |
| **AC _O2_** | Gas oxidation with 5% O_2_ at  425 ◦C, 6 h | 1281 | 149 | 0.497 | 2694 | 0 | 90 | 149 | 1321 | 4.5 |  |
| **AC _N2_** | Thermal treatment under N_2_ flow at 900 ◦C,2h | 1336 | 113 | 0.534 | 945 | 0 | 51 | 41 | 390 | n.d. | (Mezohegyi *et al.*, 2010) |
|  | Thermal treatment under N_2_ flow at 900 ◦C, 1h | 947 | 90.5 | 0.359 | 568 | 67 | 15 | 38 | 307 | 9.2 | (Pereira *et al.*, 2010, 2016) |
| **AC _H2_** | Thermal treatment under H_2_ flow at 700 ◦C, 1h | 987 | 129 | 0.377 | 341 | 48 | 0 | 11 | 249 | 10.8 | (Pereira *et al.*, 2010, 2014, 2016) |
| **ACF** | Polyacrilonitrile ACF, KoTHmex | 1098 | n.d. | n.d. | 0.78^a^ | 0.16 ^a^ | n.d. | 0.74 ^a^ | 0.23 ^a^ | 5.16 | (Amezquita-Garcia *et al.*, 2013, 2016) |
|  |  | 1099 | n.d. | 0.491 | n.d. | n.d. | n.d. | n.d. | n.d. | 4.7 | (Emilia Rios-Del Toro *et al.*, 2013) |
| **ACF _HNO3_** | Chemical oxidation with 8M of HNO_3_ at 85 ◦C | 1052 | n.d. | n.d. | 1.30 ^a^ | 0.96 ^a^ | n.d. | 0.94 ^a^ | 0.3 ^a^ | 3.41 | (Amezquita-Garcia *et al.*, 2013, 2016) |
|  | Chemical oxidation with 6M of HNO_3_ at 80◦C, 2h | 919 | n.d. | 0.419 | n.d. | n.d. | n.d. | n.d. | n.d. | 1.97 | (Emilia Rios-Del Toro *et al.*, 2013) |
| **ACF _AQDS_** | Treatment with SOCl_2_ prior to AQDS anchorage | 704 | n.d. | n.d. | 1.0 ^a^ | 3.74^a^ | n.d. | 0.81^a^ | 0 | 3.06 | (Amezquita-Garcia *et al.*, 2016) |
| **ACF _H2_** | Thermal treatment under  H_2_ atmosphere at 700 ◦C | n.d. | n.d. | n.d. | 0.625^a^ | 0^a^ | n.d. | 0.250^a^ | 1.438^a^ | 9.30 | (Amezquita-Garcia *et al.*, 2013) |
| **CXA** | Synthesized by the  sol–gel process at pH 6.25 | 540 | 168 | 0.192 | n.d. | n.d. | n.d. | n.d. | n.d. | n.d. | (Pereira *et al.*, 2014, 2016) |
| **CXB** | Synthesized by the  sol–gel process at pH 5.45 | 566 | 233 | 0.165 | n.d. | n.d. | n.d. | n.d. | n.d. | n.d. | (Pereira *et al.*, 2014, 2016) |
| **CNT** | (Nanocyl 3100) | 331 | 331 | 0 | n.d. | n.d. | n.d. | n.d. | n.d. | 7 | (Gonçalves *et al.*, 2010; Pereira *et al.*, 2014) |
|  | (Nanocyl NC3100TM) | 201 | n.d. | n.a. | n.d. | n.d. | n.d. | n.d. | n.d. | 6.6 | (Silva *et al.*, 2020, 2021) |
| **CNT _HNO3_** | Oxidation of commercial CNT with HNO_3_ (68 wt%), at boiling temperature, 14 h | n.d. | n.d. | n.d. | n.d. | n.d. | n.d. | n.d. | n.d. | 3 | (Gonçalves *et al.*, 2010; Yan *et al.*, 2014) |
|  | Oxidation of commercial CNT with 7 M HNO_3_, at boiling temperature, 3 h | 223 | n.d. | n.a. | n.d. | n.d. | n.d. | n.d. | n.d. | 2.2 | (Silva *et al.*, 2020) |
| **CNT _N2_** | Thermal treatment under N_2_ flow at 600 ◦C, 1h | 225 | n.d. | n.a. | n.d. | n.d. | n.d. | n.d. | n.d. | 6.7 | (Silva *et al.*, 2020) |
| **GO** | Graphene Supermarket ® | n.d. | n.d. | n.d. | 1.23^a^ | 1.30^a^ | n.d. | 1.26^a^ | 0.59^a^ | 2.3 | (Colunga *et al.*, 2015; Toral-Sánchez *et al.*, 2016, 2017) |
| **rGO** | GO reduction with L-ascorbic acid, at room temperature, 4h. | n.d. | n.d. | n.d. | 1.10^a^ | 0.20^a^ | n.d. | 0.21^a^ | 0.14^a^ | 7.25 | (Toral-Sánchez *et al.*, 2016, 2017) |
| **BC** | Residues from wood, slow pyrolysis 620 ◦C | 341 | n.d. | n.d. | n.d. | n.d. | n.d. | n.d. | n.d. | n.d. | (Kappler *et al.*, 2014) |
|  | Canola, pyrolysis under oxygen-low conditions at 500◦C, 4 h | 2.12 | n.d. | n.d. | n.d. | n.d. | n.d. | n.d. | n.d. | n.d. | (Tong *et al.*, 2014) |
|  | Pyrolysis under N_2_ conditions at 900 ◦C, 1 h | ~ 10.85 | n.d. | n.d. | n.d. | n.d. | n.d. | n.d. | n.d. | n.d. | (Yu *et al.*, 2015) |
| **BC _AQDS_** | Pyrolysis equilibrated with AQDS in deionized water, 72h | ~ 5.46 | n.d. | n.d. | n.d. | n.d. | n.d. | n.d. | n.d. | n.d. | (Yu *et al.*, 2015) |
| **BC__ Hydroquinone_** | BC modified by hydroquinone in the presence of oxalic acid and formaldehyde solution at 100◦C, 1 h | ~ 5.46 | n.d. | n.d. | n.d. | n.d. | n.d. | n.d. | n.d. | n.d. | (Yu *et al.*, 2015) |

^a^ The unit in this study was: meq/g; n.a. - non applicable; n.d. - non defined; S_BET_ - total specific surface area; S_meso_ - non-microporous surface area.

**Table S2. Tailored nanomaterials used as redox mediators in anaerobic remediation of pollutants: method of preparation and results of characterization**

| **Nanomaterial** | **Method** | **S _BET_**  **(m^2^ g^-1^)** | | **Total pore volume (cm^3^ g^−1^)** | **Carbon (%)** | **Metal (%)** | **Semi- metal (%)** | **Oxygen (%)** | | **pH_pzc_** | **References** |
| --- | --- | --- | --- | --- | --- | --- | --- | --- | --- | --- | --- |
| **SBC Zn _400_** | Impregnation of Zn into SCB using of ZnCl_2_ solutions, followed by carbonization at 400°C | 13.0 | 0.03 | | n.d. | 16 | n.d. | n.d. | | n.d. | (Athalathil *et al.*, 2014) |
| **SBC Zn _800_** | Impregnation of Zn into SCB using ZnCl_2_ solutions, followed by carbonization at 800°C | 202.0 | 0.24 | | n.d. | 16 | n.d. | n.d. | | n.d. |  |
| **SBC Zn _600_** | Impregnation of Zn into SCB using ZnCl_2_ solutions, followed by carbonization at 600°C | 111.3 | 0.20 | | 49.81 | 16 | 7.78^a^ | 23.05 | | n.d. | (Athalathil *et al.*, 2014, 2015) |
| **SBC Co _600_** | Impregnation of Co into SCB using CoSO_4_.7H_2_O solutions, followed by carbonization at 600°C | 161 | 0.20 | | 44.68 | 15 | 5.64^a^ | 24.2 | n.d. | | (Athalathil *et al.*, 2015) |
| **SBC Ni _600_** | Impregnation of Ni into SCB using NiCl_2_.6H_2_O solutions, followed by carbonization at 600°C | 194 | 0.24 | | 45.50 | 15 | 5.82^a^ | 21.7 | n.d. | |  |
| **SBC Fe _600_** | Impregnation of Ni into SCB using FeSO_4_.7H_2_O, followed by carbonization at 600°C | 107 | 0.15 | | 39.16 | 14 | 6.29^a^ | 29.29 | n.d. | |  |
| **rGO/Ag** | Dispersion of GO in water- ethanol solution, followed by Ag impregnation using AgNO_3_ as precursor. | n.d. | n.d. | | n.d. | 21.55 % (wt)^b^ | n.a. | n.d. | n.d. | | (Ji *et al.*, 2015) |
| **RGO/Fe_3_O_4_/Ag** | Dispersion of GO in water- ethanol solution, followed by Fe_3_O_4_ addition and Ag impregnation using AgNO_3_ as precursor | n.d. | n.d. | | n.d. | 16.44- 12.11 % (wt)^b^ | n.a. | n.d. | n.d. | |  |
| **FeO** | Co-precipitation: FeCl_3_·6H_2_O and FeCl_2_·4H_2_O dissolved in HCl solution, at room temperature | 154 | n.d. | | n.a. | 98 | n.a. | n.d. | >10 | | (Pereira *et al.*, 2017) |
| **C@FeO CVD750** | FeO coating with carbon by CVD, with ethane as carbon precursor, followed by a thermal treatment at increasing temperature, until 750◦C | 63 | n.d. | | 16 | 84 | n.a. | n.d. | >10 | |  |
| **C@FeO CVD850** | FeO coating with carbon by CVD, with ethane as carbon precursor, followed by a thermal treatment at increasing temperature, until 850◦C | 29 | n.d. | | 35 | 65 | n.a. | n.d. | >10 | |  |
| **C@FeO CVD750_·NH3_** | Thermal treatment of C@FeO CVD750 under C_2_H_6_:NH_3_ (20:10) gas mixture at 750◦C, during 2 h and then cooled under nitrogen flow until room temperature | 29 | n.d. | | 34 | 66 | n.a. | n.d. | >10 | |  |
| **MnFeO** | Co-precipitation: MnSO_4_·H_2_O and FeCl_3_.6H_2_O dissolved in HCl and water, at 50◦C | 101 | n.d. | | n.a. | 98 | n.a. | n.d. | >10 | |  |
| **C@MnFeO CVD750** | MnFeO coating with carbon by CVD, with ethane as carbon precursor, followed by a thermal treatment at increasing temperature, until 750◦C | 45 | n.d. | | 22 | 78 | n.a. | n.d. | >10 | |  |
| **C@MnFeO CVD750_·NH3_** | Thermal treatment of C@MnFeO CVD750 under C2H_6_:NH_3_ (20:10) gas mixture at 750◦C, during 2 h and then, cooled under nitrogen flow until room temperature | 31 | n.d. | | 32 | 68 | n.a. | n.d. | >10 | |  |
| **CoFeO** | Co-precipitation: Co(NO_3_)_2_·6H_2_O and FeCl_3_·6H_2_O dissolved in HCl and water, at 50◦C. | 184 | n.d. | | n.a. | 96 | n.a. | n.d. | >10 | |  |
| **C@CoFeO CVD750** | CoFeO coating with carbon by CVD, with ethane as carbon precursor, followed by a thermal treatment at increasing temperature, until 750◦C | 29 | n.d. | | 34 | 66 | n.a. | n.d. | >10 | |  |
| **C@CoFeO CVD750_·NH3_** | Thermal treatment of C@CoFeO CVD750 under C_2_H_6_:NH_3_ (20:10) gas mixture at 750◦C during 2 h and then cooled under nitrogen flow until room temperature. | 38 | n.d. | | 36 | 74 | n.a. | n.d. | >10 | |  |
| **C@FeO HdM** | FeO were coated with carbon, by HdM, with glucose as carbon precursor, followed by a thermal treatment at 180◦C | 31 | n.d. | | 39 | 61 | n.a. | n.d. | 6.7 | |  |
| **CNT@2%Fe** | CNT were impregnated with Fe using a solution of Fe(NO_3_)_3_ as precursor,. followed by a thermal treatment at 400◦C, under hydrogen flow | 266 | n.d. | | 98 | 2 | n.a. | n.d. | n.d. | |  |
|  |  | 196 | 0.440 | | n.d. | 2 | n.a. | n.d. | 6.5 | | (Silva *et al.*, 2020, 2021) |
| **CNTHNO_3_@2%Fe** | CNT were oxidized with 7 M HNO_3_, at boiling temperature, 3 h and then, impregnated with Fe | 208 | 0.444 | | n.d. | n.d. | n.a. | n.d. | n.d. | | (Silva *et al.*, 2020) |
| **CNTN_2_@2%Fe** | CNT were submitted to a thermal treatment under N_2_ flow at 600 ◦C, 1h and then impregnated with Fe | 243 | 0.581 | | n.d. | n.d. | n.a. | n.d. | n.d. | | (Silva *et al.*, 2020) |
| **CNT/AQS/Fe_3_O_4_** | Co-precipitation: FeCl_3_·6H_2_O and FeCl_2_·4H_2_O dissolved in HCl solution, at room temperature (Fe_3_O_4_). Then, CNT were placed into Fe_3_O_4_ suspension, and AQS incorporated, at 60 °C. | 14.91 | n.d. | | n.d. | n.d. | n.d. | n.d. | n.d. | | (He *et al.*, 2020) |
| **CNT/HA/Fe_3_O_4_** | Epoxy resin, Fe_3_O_4_/CNT and humic acid were added to samples of petroleum ether, followed by a thermal treatment at 180 °C | 5.88 | n.d. | | n.d. | n.d. | n.d. | n.d. | n.d. | |  |
| **RGO/Fe_3_O_4_ nanosacks** | Colloidal suspensions of GO and magnetite were assembled through aerosolphase methodology, resulting in cargo-filled graphene nanosacks | 78 | 0.496 | | n.d. | n.d. | n.a. | n.d. | n.d. | | (Chen *et al.*, 2013; Toral-Sánchez *et al.*, 2018) |
| **Fe(OH)_3_@biochar** | Fe(OH)_3_@biochar were prepared by sol-gel method, using Fe(NO_3_)_3_·9H_2_O solution as precursor | n.d. | n.d. | | 43.60 | n.d. | 3.75 | 38.06 | n.d. | | (Shi, H. Han, *et al.*, 2019) |
| **Fe(OH)_3_@PAC** | Fe(OH)_3_@PAC were prepared by sol-gel method. Fe(NO_3_)_3_·9H_2_O solution was added to PAC as precursor | 1003.66 | n.d. | | 51.61 | n.d. | 5.26 | 28.70 | n.d. | | (Li *et al.*, 2019; Shi, Y. Han, *et al.*, 2019) |

^a^ Si content; **^b^** Ag content; CVD – Carbon Vapor Deposition; Fe(OH)_3_@biochar - Fe(OH)_3_ incorporated in biochar; Fe(OH)_3_@PAC - Fe(OH)_3_ incorporated in powder AC; HdM – hydrothermal method; n.d. - non defined; n.a. - non applicable; S_BET_ - total specific surface area.
